# Intact polar lipidome and membrane adaptations of microbial communities inhabiting serpentinite-hosted fluids

**DOI:** 10.3389/fmicb.2023.1198786

**Published:** 2023-11-10

**Authors:** Kaitlin R. Rempfert, Emily A. Kraus, Daniel B. Nothaft, Nadia Dildar, John R. Spear, Julio Sepúlveda, Alexis S. Templeton

**Affiliations:** ^1^Department of Geological Sciences, University of Colorado, Boulder, CO, United States; ^2^Department of Civil and Environmental Engineering, Colorado School of Mines, Golden, CO, United States; ^3^Department of Quantitative Biosciences and Engineering, Colorado School of Mines, Golden, CO, United States

**Keywords:** serpentinization, habitability, intact polar lipids, lipid membrane adaptations, untargeted lipidomics, polyextreme conditions, Samail ophiolite, subsurface microbiome

## Abstract

The generation of hydrogen and reduced carbon compounds during serpentinization provides sustained energy for microorganisms on Earth, and possibly on other extraterrestrial bodies (e.g., Mars, icy satellites). However, the geochemical conditions that arise from water-rock reaction also challenge the known limits of microbial physiology, such as hyperalkaline pH, limited electron acceptors and inorganic carbon. Because cell membranes act as a primary barrier between a cell and its environment, lipids are a vital component in microbial acclimation to challenging physicochemical conditions. To probe the diversity of cell membrane lipids produced in serpentinizing settings and identify membrane adaptations to this environment, we conducted the first comprehensive intact polar lipid (IPL) biomarker survey of microbial communities inhabiting the subsurface at a terrestrial site of serpentinization. We used an expansive, custom environmental lipid database that expands the application of targeted and untargeted lipodomics in the study of microbial and biogeochemical processes. IPLs extracted from serpentinite-hosted fluid communities were comprised of >90% isoprenoidal and non-isoprenoidal diether glycolipids likely produced by archaeal methanogens and sulfate-reducing bacteria. Phospholipids only constituted ~1% of the intact polar lipidome. In addition to abundant diether glycolipids, betaine and trimethylated-ornithine aminolipids and glycosphingolipids were also detected, indicating pervasive membrane modifications in response to phosphate limitation. The carbon oxidation state of IPL backbones was positively correlated with the reduction potential of fluids, which may signify an energy conservation strategy for lipid synthesis. Together, these data suggest microorganisms inhabiting serpentinites possess a unique combination of membrane adaptations that allow for their survival in polyextreme environments. The persistence of IPLs in fluids beyond the presence of their source organisms, as indicated by 16S rRNA genes and transcripts, is promising for the detection of extinct life in serpentinizing settings through lipid biomarker signatures. These data contribute new insights into the complexity of lipid structures generated in actively serpentinizing environments and provide valuable context to aid in the reconstruction of past microbial activity from fossil lipid records of terrestrial serpentinites and the search for biosignatures elsewhere in our solar system.

## 1. Introduction

Efforts to detect life on other planetary bodies can be informed by investigating the distribution, diversity, and adaptations of extant life in planetary analog environments on Earth. Of particular interest are serpentinizing settings because the reducing conditions that arise during the hydration of ultramafic rock can yield abundant energy for microbial metabolism through the generation of hydrogen and organic compounds (Schulte et al., 2006; Russell et al., 2010). Both physiological and phylogenetic evidence support the hypothesis that microbial metabolisms dependent on substrates derived from serpentinization were among the first metabolisms on early Earth (Russell et al., 2010; Weiss et al., 2016; Boyd et al., 2020), and diverse microbial communities have been found to inhabit modern serpentinite-hosted fluids (Brazelton et al., 2013; Suzuki et al., 2013; Postec et al., 2015; Rempfert et al., 2017). Because ultramafic rocks are common not just in the Earth’s mantle, but also on Mars and in the cores of icy satellites, the potential for serpentinization to provide sufficient energy to support life is widespread throughout the solar system and through time (Vance et al., 2007; Quesnel et al., 2009; Sleep et al., 2011; Tarnas et al., 2018).

While extended water-rock reaction may provide ample reducing power for microbial metabolism, the geochemical conditions that result from serpentinization also pose challenges to the physiology of microorganisms and influence the distribution and composition of microbial communities in these settings (Brazelton et al., 2013; Suzuki et al., 2013, 2017; Cardace et al., 2015; Meyer-Dombard et al., 2015; Postec et al., 2015; Woycheese et al., 2015; Miller et al., 2016; Crespo-Medina et al., 2017; Rempfert et al., 2017; Twing et al., 2017; Fones et al., 2019; Sabuda et al., 2020; Seyler et al., 2020; Kraus et al., 2021). Reacted fluids are often highly reduced and hyperalkaline (pH > 11), limiting the availability of oxidants for microbial metabolism and complicating maintenance of a proton motive force across the cellular membrane (Schrenk et al., 2013). As an additional consequence of the high pH of reacted fluids, dissolved inorganic carbon and nutrients, such as phosphate, are rapidly depleted through precipitation of minerals and sorption which limits microbial carbon fixation and biosynthesis in this environment (Chavagnac et al., 2013; Schrenk et al., 2013). Microbial life capable of harnessing energy from water-rock reaction in serpentinites must adapt to these polyextreme conditions to survive.

A primary strategy microorganisms employ to adapt to extreme environments is to modify their cellular membranes, as the cell membrane plays an integral role in both shielding the cell from its environment and in preserving disequilibrium in chemical energy for metabolism (Siliakus et al., 2017). Lipids, as a bilayer or monolayer, are the primary components of the cytoplasmic membrane of microbial cells. The physical properties of lipid membranes are dependent on the chemical structures of the individual lipids that comprise the membrane, and microorganisms are capable of rapidly adjusting the composition of their lipid membrane in order to maintain membrane integrity and functionality (Benning et al., 1995; Rowlett et al., 2017; Okur et al., 2019; Chwastek et al., 2020). Membrane lipid remodeling may also be an important adaptation to reduce energetic costs of biosynthesis or the need for specific macronutrients that may be limited (Schubotz, 2019; Boyer et al., 2020). Modifications to lipids include altering the polar lipid headgroup, configuration of the backbone linking the polar head group to the hydrophilic chains, or saturation and branching of hydrophobic chains, however all adjustments require an underlying genetic capability, and consequently can be specific to taxonomic groups (Parsons and Rock, 2013; Sohlenkamp and Geiger, 2016; Siliakus et al., 2017). In polyextreme environments, the lipidome thus reflects a combination of evolved physiological adaptations to the challenging conditions, short-term regulated enzymatic remodeling in response to changing environmental parameters, and the overall microbial community composition.

Due to the recalcitrant nature of lipids compared to other biomolecules (e.g., DNA, RNA, proteins; Brocks and Summons, 2003; Walters et al., 2004; Eigenbrode, 2008), numerous studies have identified lipids in serpentinizing settings with the goal of determining signatures that can be used to trace ancient microbial activity on Earth or even to inform the search for life on extraterrestrial bodies (Klein et al., 2015; Zwicker et al., 2018; Newman et al., 2020; Rattray et al., 2022). Accordingly, studies of serpentinite-hosted lipids have focused almost exclusively on core lipid moieties that are retained in rock (Klein et al., 2015; Zwicker et al., 2018; Newman et al., 2020), and not on intact polar lipids (IPLs), which represent a more recent lipid signature, since covalently-bound headgroups are susceptible to hydrolysis after cell death (White et al., 1979; Harvey et al., 1986; Logemann et al., 2011; Sturt et al., 2004). Because IPLs reflect modern microbial communities and carry additional structural information (e.g., lipid headgroup and backbone configuration), IPLs are more suitable molecules for correlating lipid adaptations to ambient geochemistry than core lipids. A recent study by Rattray et al. (2022) reported complex IPLs in calcite and brucite veins in serpentinite rock at the Chimera Seeps in Turkey, illustrating a need to explore the conditions under which diverse lipids are produced in serpentinizing environments. To our knowledge, the IPL composition of extant biomass has only been comprehensively investigated in serpentinized fluids at one site of active serpentinization, the Lost City Hydrothermal Field, a marine hydrothermal system (~90° C) venting into oxygenated seawater (Bradley et al., 2009a,b). Inventorying the fluid-hosted lipidome of a terrestrial site of serpentinization would provide an opportunity to isolate lipid membrane modifications specific to the geochemical conditions imposed by water-rock reaction, in the absence of additional osmotic pressures and temperature. Elucidating the source and type of lipid membrane adaptations that occur in the subsurface of terrestrial serpentinizing settings would improve interpretation of preserved lipid signatures in serpentinite rock and inform potential sampling efforts for biosignature detection on other planetary bodies such as Mars.

Here, we present the first IPL survey of serpentinized fluids from a terrestrial site of low-temperature serpentinization, in the Samail Ophiolite of Oman. We examined the distribution and diversity of IPL compounds in subsurface, serpentinite-hosted fluids that spanned both a large range of pH (7.6–11.3) and reduction potential (Eh +269 to −253 mV). We developed an expansive theoretical database (> 2 million lipids) to be able to classify diverse IPLs from this setting. The intact lipidome of these fluids was characterized within the framework of aqueous geochemistry and microbial community composition, which allows us to infer source organisms for lipid signatures and identify potential membrane modifications to improve understanding of the adaptations that enable microbial life to inhabit this polyextreme environment.

## 2. Materials and methods

### 2.1. Site description and sampling of subsurface fluids

In February 2017, subsurface fluids were pumped from six preexisting wells previously drilled into the crust–mantle section of the Samail Ophiolite by the Oman Ministry of Regional Municipalities and Water Resources (Table 1).

**Table 1:** Locations of wells and borehole sampling parameters.

Well	WAB188	WAB105	WAB104	WAB55	WAB71	NSHQ14
Lithology	gabbro	peridotite	peridotite	peridotite	peridotite	peridotite
UTM Easting	671,123	644,678	643,099	634,777	670,322	675,495
UTM Northing	2,529,798	2,536,524	2,541,124	2,506,101	2,533,981	2,529,716
elevation (masl)	514	738	842	531	608	526
well depth (m)	78	120.5	120.4	102	136.5	304
screened interval (mbc)	34.5–51	110–117	100.8–104	8–97	128–131	open below casing
depth to water (mbc)	9.5	16.2	35	7.7	7.7	10^a^
pump depth (mbl)	78	50	28	26	50	85
L filtered for lipid analysis	16.3	162	9.9	115.9	20.2	67.3

^a^ indicates data from 2017 was not available and replaced with 2016 data published by Rempfert et al. (2017).

At each well, a Grundfos SQ2-85 submersible pump (Grundfos Pumps Corp., Denmark, Netherlands) attached to a splitting manifold and Teflon tubing was utilized to collect water at depth (Table 1). The pump, manifold, tubing, and filter housings were flushed with site water for 20–30 min (~100 L of water) prior to sampling. Biomass was concentrated for lipid analysis on combusted (450° C, 8 h) 0.3 μm Advantec (Advantec MFS, Inc., Dublin, CA) glass fiber filters in a Millipore 47 mm stainless steel housing (Millipore Sigma, Burlington, MA), and for DNA/RNA analysis on 0.2 μm Millipore polycarbonate filters in a 47 mm Pall (Pall Corporation, Cortland, NY) polycarbonate filter housing. The volume of water filtered at each well was measured by collecting the filtrate in a graduated cylinder. Biomass for DNA/RNA analysis was suspended in bead tubes with lysis/stabilization solution (Zymo Research Inc., Irvine, CA) and frozen in a liquid nitrogen dewar on site. Glass fiber filters with concentrated biomass for lipid analysis were placed in combusted aluminum foil and frozen inside sterile cryovials until analysis. Measured volumes of well water filtered for lipid analysis are listed in Table 1.

Water temperature, conductivity, pH, and oxidation–reduction potential (Eh) were measured in the field with a Hach HQ40D Portable Multi Meter (Loveland, CO). Aqueous phase gas sampling was conducted using the “bubble strip” method (modified from Kampbell et al., 1998, Nothaft, 2019). Additionally, filtered well water (passed through the 0.22 μm filter) was collected in 15 mL Falcon^®^ tubes (Corning Inc., Corning, NY) for quantification of major anions and cations, with the latter acidified with nitric acid in the field at the time of collection to a final pH <2. Aliquots for dissolved inorganic carbon quantification were injected through 0.22 μm polyethersulfone Basix syringe filters (Thermo Fisher Scientific, Waltham, MA) into butyl-stoppered vials that had previously been evacuated, acid-washed, and combusted.

### 2.2. Aqueous geochemical analyses

Protocols for aqueous geochemical analyses are described in detail in Kraus et al. (2021). Briefly, dissolved H_2_ and CH_4_ were measured using an SRI 8610C gas chromatograph (SRI instruments, Torrance, CA) with a 2 × 1 mm ID micropacked ShinCarbon ST column (Agilent, Santa Clara, CA) and N_2_ as the carrier gas. Peak intensities were measured on a thermal conductivity detector for H_2_ and a flame ionization detector for CH_4_. Peak intensities were calibrated (±2%) with standard gas mixes (Supelco Analytical, Bellefonte, PA) with a relative standard deviation of ~5% over the calibrated range. Concentrations of cations and anions were measured via inductively coupled plasma atomic emission spectroscopy (ICP-AES; Optima 5,300, Perkin-Elmer, Fremont, CA) and ion chromatography (IC; ICS-90; Dionex, Sunnyvale, CA) at the Colorado School of Mines. For DIC analyses, 6 mL aliquots of samples were transferred to helium-purged Exetainer^®^ tubes (Labco, Ceredigion, United Kingdom) and converted to CO_2_ for analysis by addition of boiled 85% phosphoric acid (H_3_PO_4_). Equilibrated and converted samples were then quantified using a Delta V Isotope Ratio Mass Spectrometer equipped with a Thermo Gasbench II gas preparation and introduction system (Thermo Fisher Scientific) at the Earth Systems Stable Isotope Laboratory at the University of Colorado, Boulder.

### 2.3. DNA and RNA extraction, sequencing, and processing

Prior to extraction, cells were lysed by bead beating in one-minute intervals for a total of 5 mins (with one-minute rests between beating intervals to cool the sample tubes to prevent sample degradation). DNA and RNA were then extracted in parallel using the Zymo microbiomics soil/fecal DNA miniprep extraction kit (Zymo Research Inc.) according to manufacturer instructions. DNA was quantified by the Qubit double-stranded DNA high-sensitivity assay (ThermoFisher Scientific) and then frozen at −80°C. Extracted RNA was first converted to cDNA through reverse transcription-PCR as described previously (Kraus et al., 2021) and then quantified and stored frozen.

SSU rRNA genes were amplified from both DNA and cDNA using the 515-Y M13 and 926R primer set (Parada et al., 2016; Kraus et al., 2018) which spans the V4 and V5 hypervariable regions. PCR conditions and barcode reactions utilized in this study were described by Kraus et al. (2018). Final products were purified with Pure beads (Kapa Biosystensm Wilmington, MA) and pooled in equimolar amounts before concentration to a final volume of 80 μL on a Ultracel-30 K membrane (Millipore Sigma) within an Amicon Ultra 0.5 mL centrifugal filter (Millipore Sigma). The prepared DNA/cDNA library was sequenced using V2 PE250 chemistry on an Illumina MiSeq sequencer (Illumina Inc., San Diego, CA) at the Duke Center for Genomic and Computational Biology.^1^ All raw sequences are available under accession PRJNA560313 on the NCBI Sequence Read Archive.

Sequence files were demultiplexed and trimmed using Cutadapt (Martin, 2011) and quality filtered using Figaro v1.1.1.^2^ Amplicon sequence variants (ASVs) were then identified in the “DADA2” R package (Callahan et al., 2016) and assigned taxonomy to the genus level using the RDP classifier (Wang et al., 2007) trained on the Silva SSU 138 reference database (Quast et al., 2013). Sequences assigned to mitochondria, chloroplasts, eukaryotes, or not assigned at the domain level (collectively <1% of sequences), were removed. Processing scripts are available online^3^ under folder “OM17.”

### 2.4. Lipid extraction and analysis

Intact polar lipids were extracted from glass-fiber filters loaded with biomass using a modified (Wörmer et al., 2015) Bligh and Dyer method (Bligh and Dyer, 1959). Samples were subjected to a total of five sequential extractions by ultrasonication. Two extractions were performed using 2:1:0.8 v:v:v methanol/dichloromethane/phosphate buffer (50 mM dipotassium phosphate monobasic, adjusted to pH 7.4), followed by two extractions in 2:1:0.8 v:v:v methanol/dichloromethane/TCA buffer (5% trichloroacetic acid, adjusted to pH 2), and a final extraction in 5:1 v:v methanol/dichloromethane. Prior to extraction, 200 ng of C16-PAF (Avanti Polar Lipids, Inc., Alabaster, Alabama) was added to each sample as an extraction standard to assess yield. Entire glass fiber filters were extracted using 4 mL of extraction buffer per extraction step. Supernatant from each extraction step was pooled in a separatory funnel. We separated and collected the organic fraction of the total lipid extract (TLE) after addition of 1:1 v:v dichloromethane/water in an amount equal to the total buffer utilized throughout the combined extraction steps. TLEs were then concentrated under a gentle flow of nitrogen gas (UHP grade) using a turbovap^®^ evaporator, filtered through a 0.45 μm polytetrafluoroethylene syringe filter, re-dissolved in 100 uL of 9:1 v:v dichloromethane/methanol, and transferred to a 2 mL vial with insert prior to analysis. 1 ng of deuterated standard (d9-DGTS, Avanti Polar Lipids) was added to each insert to correct for sample matrix effects on ionization and to monitor retention times between samples.

Lipid extracts were analyzed by high performance liquid chromatography with heated electrospray ionization high resolution mass spectrometry (HPLC-HESI-HRMS) on a Thermo Scientific UltiMate 3000 system coupled to a Q Exactive Focus hybrid Quadrupole-Orbitrap mass spectrometer at the Organic Geochemistry Lab in the University of Colorado, Boulder. Untargeted screening of all samples was performed in full scan coupled to TopN data-dependent MS–MS mode (full scan-ddMS2) with dual positive and negative ionization in which a full scan of parent ions across the entire mass range was obtained and the top three parent ions at any given time were successively isolated and fragmented in the next scan. Separation of IPL headgroups was achieved using hydrophilic interaction liquid chromatography (HILIC) under conditions described by Wörmer et al. (2013) on an Acquity BEH Amide column (1.7 μm, 2.1 by 150 mm column, Waters Corporation, Eschborn, Germany). Eluent A consisted of 75:25 v:v acetonitrile/dichloromethane with 0.01% formic acid and 0.01% ammonium hydroxide; eluent B consisted of 50:50 v:v methanol/water with 0.4% formic acid and 0.4% ammonium hydroxide. Gradient elution was performed at a constant flow rate of 0.4 mL/min under the following gradients: 1% B for 2.5 min, 1 to 5% B from 2.5 to 4 min, 5 to 25% B from 4 to 22.5 min, 25% B to 40% B from 22.5 to 26.5 min, held at 40% B from 26.5 to 27.5 min, dropped to 1% B from 27.5 to 28.5 min and then held at 1% B to re-equilibrate the column until the end of the run (total run time 50 min). The column was kept at 40°C throughout the duration of each run, and 10 μL of sample dissolved in 9:1 v:v dichloromethane/methanol was injected per run. Optimal electrospray source parameters for ionization of lipid classes as either hydrogen or ammonium adducts with positive ionization, or formate or deprotonated adducts with negative ionization, were used: spray voltage of 3.5 kV, sheath gas flow of 40 arbitrary units (AU), auxiliary gas flow of 5 AU, S-lens RF level of 55 AU, capillary temperature of 200°C, and auxiliary gas heater temperature of 250°C. A scan range of 400–2000 m/z was used and mass resolution was set to the maximum possible value of 70,000 (FWHM at 200 m/z) for full-scan and 17,500 for MS2, with an AGC target of 1e5, minimum AGC target of 1e4, 200 ms maximum injection time, stepped collisional energy (nce: 10, 30, 70), and 3 m/z isolation window for MS2 scans.

The mass spectrometer was calibrated for mass resolution and accuracy weekly through direct infusion of Pierce LTQ Velos ESI Positive (88323) and Negative (88324) Ion Calibration Solution (ThermoFisher Scientific). Real-time mass accuracy and correction was performed using the lock mass of a low-level eluent contaminant polysiloxane (391.28429).

### 2.5. Generation of theoretical intact polar lipids database

We developed a custom *in silico* IPL database for environmental lipids by adapting “LOBSTAHS” (Collins et al., 2016), an existing bioinformatic software package in R (R Core Team, 2020). We modified the LOBSTAHS “generateLOBdbase.R” script to allow for the addition of mixed acyl/ether glycerol (AEG), monoether glycerol (MEG), diether glycerol (DEG), ceramide (Cer), 1,2 alkanediol (AD), and fatty amide (FA) backbone structures in addition to the diacyl glycerol (DAG) and monoacyl glycerol (MAG) backbones already included in the package structure. Additionally, we increased the number of allowed chains from 2 to 4 to include cardiolipins and triglyceride lipids. Possible headgroups were expanded through compilation of previously identified headgroup structures from environmental IPLs from published literature. Chemical formulas and references (Kates, 1993; Benning et al., 1995; Karlsson et al., 1998; Ferreira et al., 1999; Kawahara et al., 2002; Sturt et al., 2004; Yang et al., 2006, 2010; Zhang et al., 2009; Murphy and Axelsen, 2011; Rossel et al., 2011; Moore et al., 2013; Popendorf et al., 2013; Diercks et al., 2015; Schubotz et al., 2013, 2015, 2018; Lobasso et al., 2015; Moore et al., 2015, 2016; Wang et al., 2015; Wörmer et al., 2015; Yoshinaga et al., 2011, 2012, 2015; Becker et al., 2016; Bosak et al., 2016; Hewelt-Belka et al., 2016; Li et al., 2017; Slavetinsky et al., 2017; Luo et al., 2018; Bale et al., 2019; Boyer et al., 2020; Tsugawa et al., 2020) for included headgroups are reported in Supplementary Table S1.

For generation of the database, the summed formulas of headgroup and backbone combinations (Supplementary Table S1) were inputted as separate entries in the LOBSTAHS-formatted component table for iterative calculation. Additionally, formulas for the full IPL structure of ladderane and isoprenoidal diether and tetraether lipids were entered as unique components to the table. For database components calculated iteratively, we used parameters specified for lipid chains as described by Foster et al. (2013). Chains were allowed to vary in length from 2 to 30 carbons, and double bonds were allowed to vary from 0 to 6 per chain (with a minimum spacing of “3n + 2,” where n is any non-negative integer which fits within the length of the chain). Chains were also allowed to have up to 3 hydroxylations, as reported in previous studies (Smułek et al., 2015; Nowak et al., 2016).

For unique components, we used custom R scripts to combine headgroup and core (backbone + chains) formulas. These include archaeol (1–8 unsaturations, 0–2 hydroxylations, 0–1 methylations, 0–2 extensions and 0–1 abridgements of an isoprene unit), glycerol dialkyl glycerol tetraethers (GDGTs) of both branched (br-GDGTs; 0–14 methylations) and isoprenoidal varieties (0–12 double bond equivalents, 0–2 methylations, 0–2 unsaturations) and the glycerol dialkyl diether (GDD) modification of these structures, as well as ladderane lipids with 3 and 5 cyclobutane rings and 18–22 carbons in the ladderane chain. The final database contained 91 headgroups corresponding to 2,139,073 unique ionized IPLs. Custom scripts and modified LOBSTAHS scripts for database generation are publicly available.^4^

### 2.6. Identification of intact polar lipids

Intact polar lipid datafiles in the raw Thermo file format were first converted to mzXML files using the software msConvert in centroid mode according to vendor format (Adusumilli and Mallick, 2017). The resulting mzXML files were read into R (R Core Team, 2020) using the package “MSnbase” (Gatto and Lilley, 2011; Gatto et al., 2021). Peak detection, grouping, and alignment was performed using the package “xcms” (Smith et al., 2006; Tautenhahn et al., 2008; Benton et al., 2010) with parameters optimized for the dataset in the package “IPO” (Libiseller et al., 2015). The package “CAMERA” was then utilized to aggregate peak groups into pseudospectra and annotate secondary isotopic features in the dataset (Kuhl et al., 2012). Peak groups were screened preliminarily with our custom environmental IPL database in “LOBSTAHS” (Collins et al., 2016). MS2 spectra were extracted for peak groups using the “featureSpectra” function in “xcms” and spectra across samples pertaining to the same peakgroup were combined to a composite MS2 spectra using “combineSpectra.”

Only peak groups that had corresponding MS2 data for confirmation of “LOBSTAHS” identifications were included for downstream analysis. Due to the size of the custom database, multiple isomers exist for any given lipid of interest. These isomers represent different lipid structures in either core chain, backbone, or headgroup composition, but the same chemical formula, and thus measured mass on the Orbitrap. While the HILIC chromatography we employed separated lipid classes according to the polarity of their polar headgroup, we found that retention time screening by headgroup class did not entirely resolve isomer identification. Chain length and hydroxylation of core chains can result in retention time shifts of a few minutes, well within the range of typical separation between headgroup classes. We used a custom helper script to putatively annotate diagnostic MS2 fragments and neutral losses (diagnostic masses listed in Supplementary Table S2) and then manually screened annotations before final assignment of lipid identity. Positively ionized MS2 spectra were used to characterize headgroup composition, and negatively ionized MS2 were utilized to probe core lipid structure, as ester-linked fatty acids are lost from the intact structure at the collisional energies induced in our experimental method.

Identified compounds are reported as composite formulas in the format of “(headgroup abbrv.)- (backbone abbrv.) (# of carbons):(# of unsaturations) + (# of hydroxylations)O,” e.g., “PG-DAG 34:0 + 1O” for a phosphatidylglycerol diacylglycerol with two saturated chains adding up to 34C in total, with one chain hydroxylation. Because the ionization response of IPLs was poorer for negative adducts, many IPLs with diagnostic positive MS2 spectra did not have complimentary negative ion data. For this reason, and because diether lipids diagnostically lack fragmentation as negative adducts, we did not specify their individual chain lengths and properties (see Supplementary Datasheet 1 for further elaboration on assumptions for DEG assignments). In the event that no corresponding negative MS2 data existed for any lipid structure that had been putatively annotated as one of multiple structural isomers of the same headgroup, an assignment was made to the linkage of a nearby (retention time < 15 s) lipid compound of the same headgroup with negative ion confirmation, or if that was not present, to the structural isomer that did not require hydroxylation.

Raw spectrometry files are available in the MetaboLights database (Haug et al., 2020) under study identifier MTBLS7570 and processing scripts and parameters are available.^5^

### 2.7. Quantification of intact polar lipids

Prior to quantification, annotated lipids found in solvent blanks were subtracted from sample data (Broadhurst et al., 2018). Only lipids with peak areas 10-fold greater than blank averages were considered for downstream analysis. To account for any sample matrix effects on ionization efficiency, we corrected all peak areas in each sample with the ratio of the peak response for the deuterated reference compound d9-DGTS spiked as an internal standard compared to the peak response of the pure compound. Following matrix factor correction, IPL concentrations were calculated through application of analytical response factors from 31 commercially-available IPL external standards (Avanti Polar Lipids, see Supplementary Table S3). Response factors were estimated by taking the linear slope of the injected masses vs. the integrated peak areas across a 5-point dilution series (0.1, 1.0, 2.5, 5.0, 10.0 ng on column; see Supplementary Figure S1). Because authentic standards are not available for every IPL structure present in nature, or even headgroup, IPL analysis is only considered semi-quantitative. When no authentic standard was available, we assigned response factors on the basis of similarity of headgroups to existing standards, and for lipid classes with multiple measured standards, the average response factor for that class was applied. All aminolipids (e.g., OL, 3Me-OL, DGCC) were assigned to the DGTS response factor, all IPL glycolipids with nitrogen-bearing groups (e.g., NAcG-G, NAcG-P, G-GA) were assigned to the DG-Cer standard, and all glycolipids with more than one glycosyl group (e.g., GAc-G) to the DG-DAG standard.

### 2.8. Calculations and statistical analyses

Abundance-weighted properties of IPLs were calculated according to Boyer et al. (2020) using the following equation:


$$\Xi =\frac{\sum_{i}\Xi_{ipl,i}\cdot x_{i}}{\sum_{i}n_{\mathrm{component},i}\cdot x_{i}}$$

where Ξ represents the average property of interest (e.g., number of aliphatic carbons, number of unsaturations), Ξipl,i represents the property summed across all components in the ith IPL (e.g., 36 carbons in the alkyl chains of a 36:0 DAG) with ncomponent,i instances (e.g., 2 alkyl chains in a DAG), and xi represents the mole fraction of the ith IPL.

The oxidation state of carbon (Z_c_) was calculated for IPLs and their component parts (e.g., headgroup, backbone, combined core chains) using the equation:


$$Zc=\frac{2o+3n-5p-4s-h+Z}{c}$$

where Z indicates the net charge and c, h, n, o, p, and s are the number of atoms of carbon, hydrogen, nitrogen, oxygen, phosphorus, and sulfur, respectively, in the chemical formula. Abundance-weighted properties and Zc calculations were conducted using scripts provided^6^ by Boyer et al. (2020).

To correlate taxa abundances with IPLs, Pearson’s correlations of the relative abundance matrices of DNA and IPLs were calculated with Bonferroni-corrected *p*-values according to Probst et al. (2020) with modifications to the scripts provided^7^ to allow for more than one assignment for each IPL to potential source organism.

To investigate lipid distribution in relation to aqueous geochemistry, constrained analysis of principle coordinates (CAP) was performed using the “capscale” function in the R package vegan (Dixon, 2003) on the Gower dissimilarity matrix of Hellinger-transformed IPL compound relative abundances. A variation inflation factor (VIF) was calculated to ensure that there was no significant multicollinearity (VIF <10) of constrained parameters for the CAP analysis. The significance level in the CAP model was assessed by 500 data permutations.

## 3. Results

### 3.1. Geochemical characterization of subsurface fluids

Subsurface fluids were sampled from a series of six government monitoring wells previously drilled into crustal gabbro and mantle peridotite bedrock in the Samail Ophiolite, Sultanate of Oman. The chemical characterization of each well is summarized in Table 2. The pH of recovered fluids varied from 7.6 to 11.3. Hyperalkaline (pH >10) fluids sampled from wells NSHQ14 and WAB71 were also characterized by negative oxidation–reduction potentials (Eh) and low (<0.2 mM) concentrations of dissolved inorganic carbon (DIC). Potential electron acceptors were limited in these fluids, with measured sulfate and nitrate concentrations of or below 42 μM and 2.5 μM, respectively. Conversely, alkaline fluids (pH <10) hosted in the peridotite wells WAB104, WAB105, and WAB55 exhibited positive Eh values, oxidant concentrations an order of magnitude higher (sulfate ≥292 μM, nitrate ≥118 μM) than hyperalkaline fluids, and DIC concentrations up to 3 mM. WAB188, the only sampled well hosted within gabbro, was characterized by the highest concentration of sulfate (1.13 mM), and greater concentrations of potential reductants such as hydrogen and methane (0.99 μM H2, 1.8 μM CH4) compared to alkaline fluids hosted in peridotite. While dissolved aqueous-phase methane was detected in every fluid sampled, the greatest concentrations were measured in hyperalkaline fluids (14.8–106 μM). Hydrogen was only detected in hyperalkaline fluids and fluids hosted within gabbro, and was highest in concentration (253 uM) in well NSHQ14. Ammonium concentrations were highest (130 uM) in hyperalkaline fluids in well WAB71. Phosphate was below the detection limit (5.26 μM) in all fluids.

**Table 2:** Geochemical composition of sampled fluids in the 2017 field season previously reported by Kraus et al. (2021).

Well	WAB188	WAB105	WAB104	WAB55	WAB71	NSHQ14	LOQ (μM)
pump depth (mbl)	78	50	28	26	50	85	-
L filtered for lipid analysis	16.3	162	9.9	115.9	20.2	67.3	-
pH	7.6^a^	8.3^a^	8.5	9.2	10.6	11.3	-
Eh (mV)	214	178^a^	180^a^	269	−133	−253	-
SO_4_^2−^ (μM)	1.13E+03	2.92E+02	4.77E+02	8.75E+02	4.20E+01	2.00E+00	1.04E+00
NO_2_^−^ (μM)	6.00E+00	BLOQ	BLOQ	8.00E+00	1.40E+01	1.60E+01	2.17E+00
NO_3_^−^ (μM)	1.18E+02	1.35E+02	1.23E+02	1.43E+02	2.50E+00	BLOQ	1.61E+00
NH_4_^+^ (μM)	BLOQ	BLOQ	6.41E+00	BLOQ	1.00E+02	1.30E+01	1.00E+00
PO_4_^3−^(μM)	BLOQ	BLOQ	BLOQ	BLOQ	BLOQ	BLOQ	5.26E+00
H_2_ (μM)	9.92E-01	BLOQ	BLOQ	BLOQ	5.92E-01	2.53E+02	4.80E-02
CH_4_ (μM)	1.83E+00	2.01E-02	2.30E-02	1.06E-01	1.48E+01	1.06E+02	1.53E-02
DIC (mM)	3.00E+03	3.50E+03	3.50E+03	2.90E+03	1.20E+02	1.30E+02	2.00E+01
Na (total) (μM)	3.49E+03	5.92E+02	6.58E+02	4.12E+03	4.95E+03	1.02E+04	5.85E+00
Ca (total) (μM)	1.33E+03	2.69E+02	1.03E+02	5.40E+01	4.07E+03	4.34E+03	1.60E-01
Mg (total) (μM)	1.44E+03	1.69E+03	1.91E+03	2.75E+03	2.00E+00	2.00E+01	5.90E-02
K (total) (μM)	3.92E+01	2.72E+01	2.95E+01	2.10E+02	2.51E+02	2.45E+02	8.26E-01
Al (total) (μM)	8.00E-01	8.00E-01	1.00E+00	BLOQ	1.80E+00	2.00E+00	7.60E-01
Fe (total) (μM)	4.00E-01	5.00E+00	2.00E+00	2.50E+00	1.57E-01	2.00E+00	6.00E-03
Si (total) (μM)	3.69E+02	2.67E+02	1.22E+02	3.00E+00	2.10E+01	6.00E+00	4.00E-01
Cl^−^ (μM)	5.04E+03	8.55E+02	8.01E+02	7.24E+03	1.16E+04	1.62E+04	2.82E+00
Br^−^ (μM)	2.00E+00	BLOQ	BLOQ	5.00E+00	1.20E+01	2.50E+01	1.25E+00

^a^ indicates data from 2017 was not available and replaced with 2016 data published by Rempfert et al. (2017); BLOQ indicates the parameter was below the limit of quantification (limit indicated in column LOQ).

### 3.2. Custom intact polar lipid database generation

**Figure 1:**
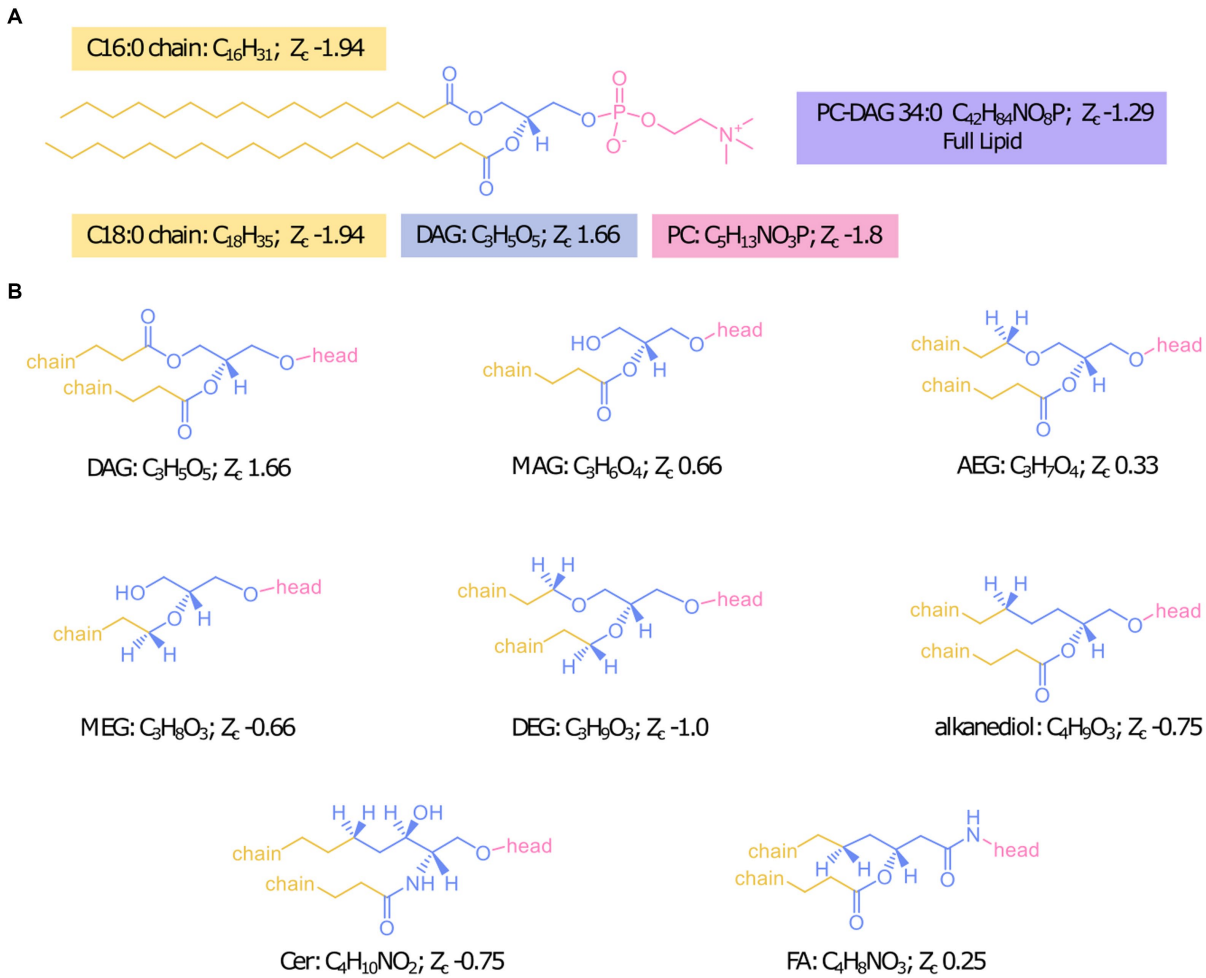
Designation of headgroup, backbone, and chains for Zc calculations with the example IPL PC-DAG 34:0 **(A)** and structures of all backbone linkages investigated **(B)**.

To be able to probe the diversity of IPL structures anticipated in this geologic setting, we developed a custom *in silico* IPL database for environmental lipids by adapting the existing bioinformatic software package “LOBSTAHS” (Collins et al., 2016). The LOBSTAHS package was developed in part to calculate the monoisotopic mass for theoretical lipid structures through user-specified combinations of IPL headgroups, acyl chain lengths, unsaturations, and hydroxylations for acylglycerol lipids. However, because this package was designed to annotate oxylipins of marine algae, the default database is limited to a few common marine lipid headgroups with ester-linkages of aliphatic chains to the lipid backbone. We expanded possible backbone configurations to include mixed acyl/ether glycerol (AEG), monoether glycerol (MEG), diether glycerol (DEG), ceramide (Cer), 1,2 alkanediol (AD), and fatty amide (FA) backbone structures in addition to the diacyl glycerol (DAG) and monoacyl glycerol (MAG) backbones already included in the package structure (Figure 1). Additionally, the number of allowed chains was increased from 2 to 4 to include cardiolipins and triglyceride lipids. We also expanded the headgroups considered by compiling previously identified headgroup structures of environmental IPLs from published literature; the chemical formulas and references for 91 included headgroups are reported in Supplementary Table S1. The final database consisted of over 2 million lipid species.

### 3.3. Inventory of intact polar lipids

**Figure 2:**
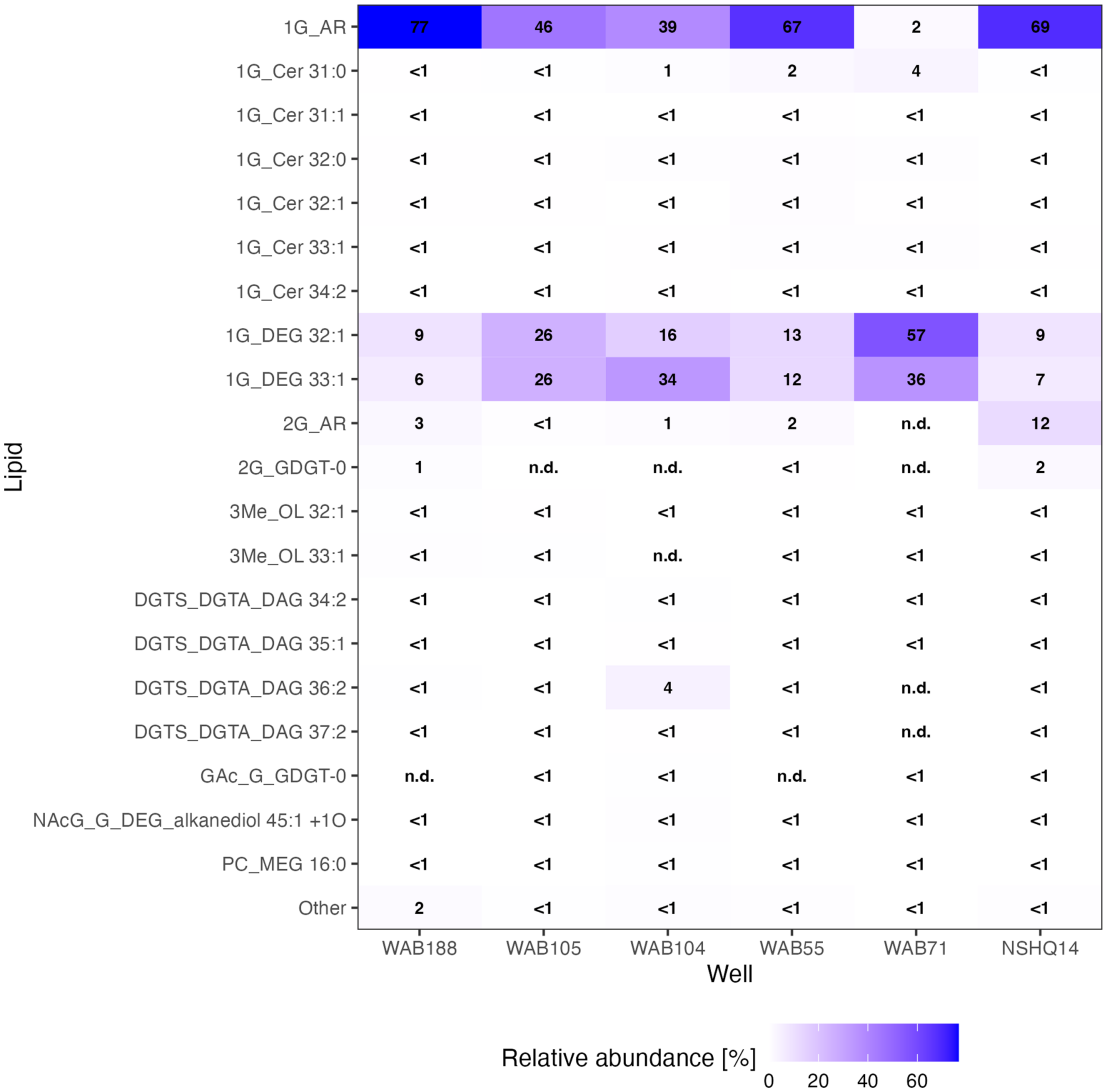
Relative abundance heatmap of the 20 most abundant intact polar lipid (IPL) compounds detected.

We were able to identify a diversity of IPLs with varying headgroup and backbone structures in serpentinite-hosted fluids using our custom environmental IPL database coupled with data-dependent MS2 screening. A total of 96 IPLs were identified; the most abundant 20 IPL compounds across all sampled wells are presented in a relative abundance heatmap in Figure 2 (see Supplementary Table S4). Notably, these 20 IPLs constituted ≥98% of the observed IPL diversity across sampled fluids.

Total IPL concentrations (normalized by liter of filtered well water) showed no trend with planktonic cell abundances previously reported for these wells from paraformaldehyde-fixed samples collected at the time of lipid sampling (Fones et al., 2019). For example, the maximum concentration of polar lipids was observed in hyperalkaline well NSHQ14 where the lowest cell abundance (1.16 × 10^5^ cells mL^−1^) was reported (Supplementary Figure S2).

**Figure 3:**
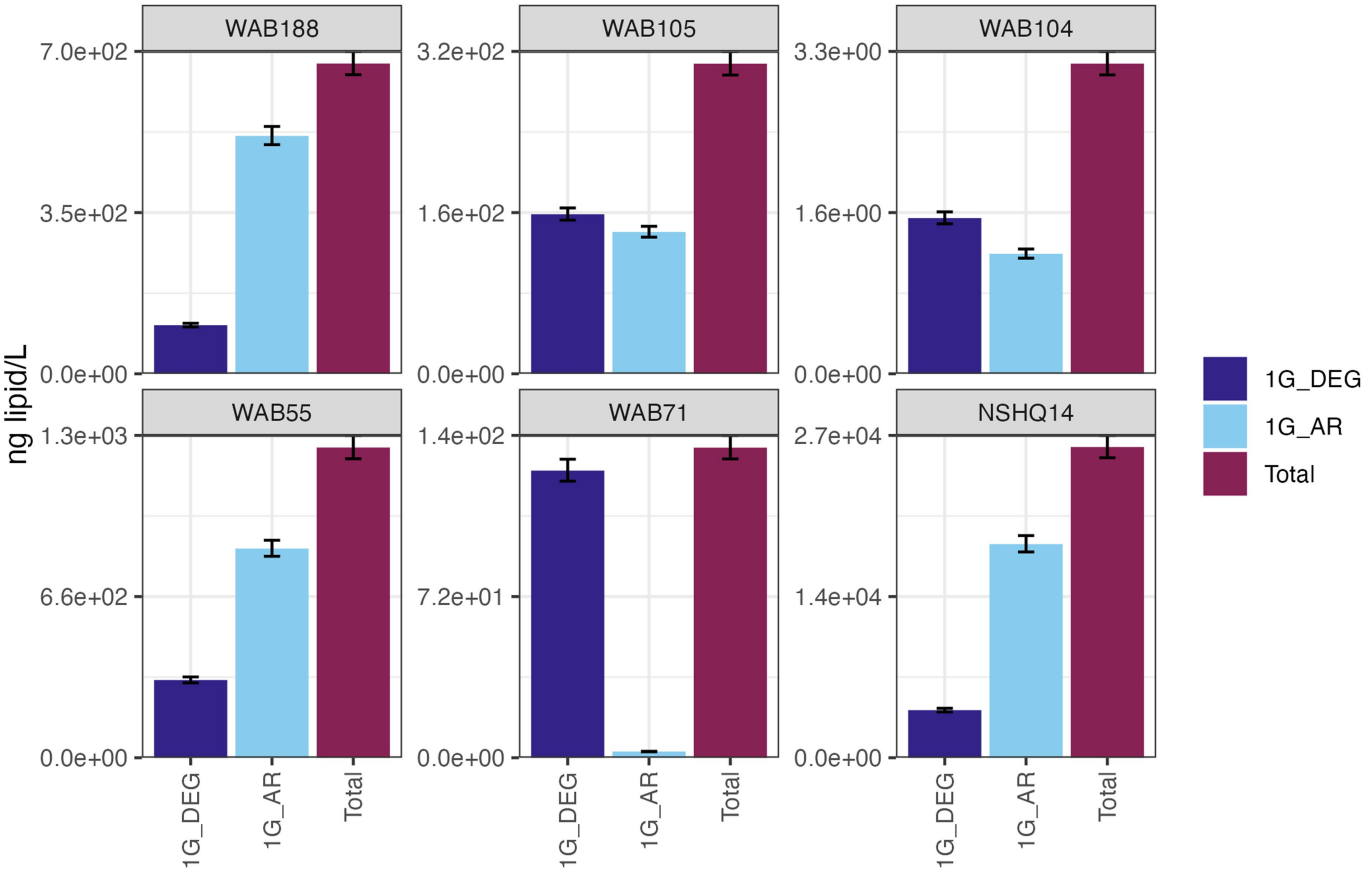
The abundances (ng of lipid/L) of bacterial non-isoprenoidal monoglycosyl diether (1G-DEG) lipids and the archaeal isoprenodial monoglycosyl diether lipid, archaeol (1G-AR), in comparison with total lipid abundances in each well.

IPLs for both Bacteria (non-isoprenoidal) and Archaea (isoprenoidal) were dominated by glycolipids, which comprised 91 to 99% of the total intact lipidome (Figure 3). Apart from well WAB71, the most abundant glycolipid in subsurface fluids was monoglycosyl archaeol (1G-AR). This archaeal diether lipid made up 77% of measured IPLs in well WAB188 and 69% of IPLs in well NSHQ14. Bacterial monoglycosyl diethers (1G-DEGs) were prevalent in all fluids with the greatest relative abundance observed in WAB71 where 93% of IPLs could be attributed to just two 1G-DEG compounds (1G-DEG 32:1 and 1G-DEG 33:1; Figure 2). In all other wells, the relative abundance of 1G-DEG lipids varied from 15 to 52%.

**Figure 4:**
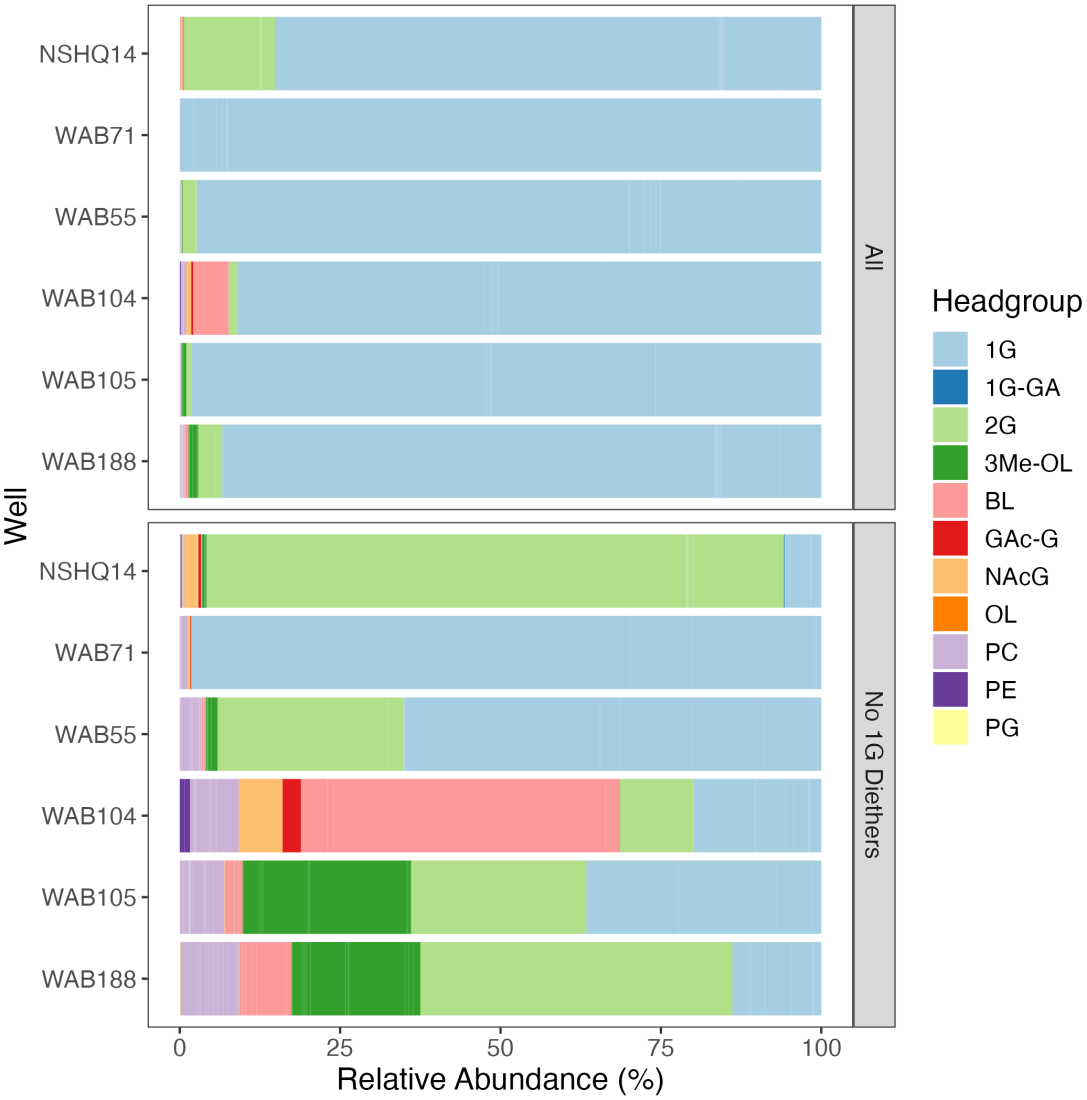
Relative abundances of IPLs by headgroup type with and without bacterial and archaeal monoglycosyl diether lipids included. Abbreviations: 1G, monoglycosyl; 1G-GA, monoglycosyl glycuronic acid; 2G, diglycosyl; 3Me-OL, trimethylated ornithine; BL, betaine lipid (DGTS and DGCC); GAc-G, acetylglycosyl monoglycosyl; NAcG, N-acetyl glycosaminyl (both NAcG-P and NAcG-G); OL, ornithine lipid, PC, phosphatidylcholine; PE, phosphatidylethanolamine; PG, phosphatidylglycerol.

Altogether, the combined relative abundance of bacterial and archaeal monoglycosyl diether lipids constituted 89 to 98% of the measured polar lipids (Figure 4), with the remaining glycolipids consisting of either glycosphingolipids (up to 4% in WAB71; primarily 1G-Cer, but 2G-Cer was present at <1% relative abundance), monoglycosyl glycoronic acid diacylglycerol (1G-GA-DAG) lipids (<1% relative abundance in NSHQ14), or diglycosyl isoprenoidal lipids (up to 14% relative abundance in NSHQ14). Membrane-spanning archaeal tetraethers were not detected in high abundance but were observed primarily in well NSHQ14 where 2G-GDGT-0 was present at 2% relative abundance.

Phospholipids were present at less than 1.1% relative abundance in all wells. Due to the predominance of archaeal and bacterial 1G-diether lipids, the lower barplot in Figure 4 displays the relative abundances of minor lipids (<12%) excluding these major lipid classes. Phospholipid classes included phosphocholine (PC) diacylglycerol (DAG), dietherglycerol (DEG), monoetherglycerol (MEG) and mixed acyl/ether glycerol (AEG) lipids, phosphoethanolamine sphingolipids (PE-Cer), and phosphatidylglycerol diacylglycerol (PG-DAG) lipids. Aminolipids were relatively more abundant than phospholipids, comprising up to 5.5% relative abundance of detected IPLs. Betaine lipids were particularly abundant in well WAB104 where diacylglyceryl-trimethylhomoserine (DGTS)-DAG 36:2 constituted 4% of the lipidome. Ornithine lipids (OL), particularly trimethylated ornithine (3Me-OL) lipids, were detected in all fluids with the greatest relative abundance in WAB105 and WAB188, where cumulatively this class made up 0.7 and 1.5% of the lipidome, respectively. In addition, two classes of lipids with N-acetylglucosaminyl (NAcG) headgroups and diether backbones were detected at <1% combined relative abundance across fluids.

### 3.4. Correlation of intact polar lipidome with potential source organisms

**Figure 5:**
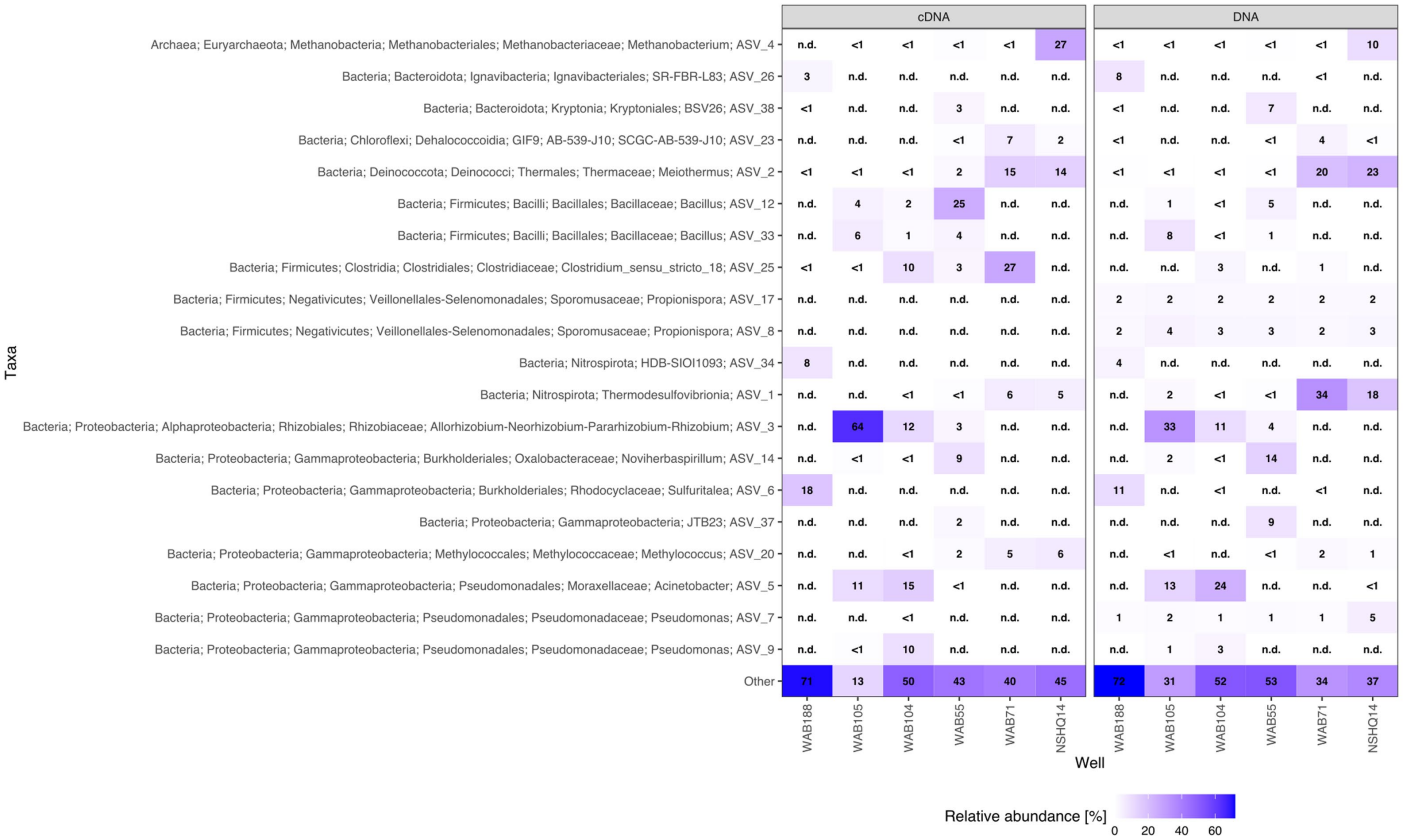
Relative abundance heatmap of top 20 ASVs for cDNA and DNA.

We coupled lipidomic analyses with 16S rRNA gene amplicon sequencing of DNA and RNA transcripts (RNA converted to cDNA) generated from biomass collected from the same fluids at the time of IPL sampling to infer potential source organisms for observed IPL compounds (Figure 5).

**Figure 6:**
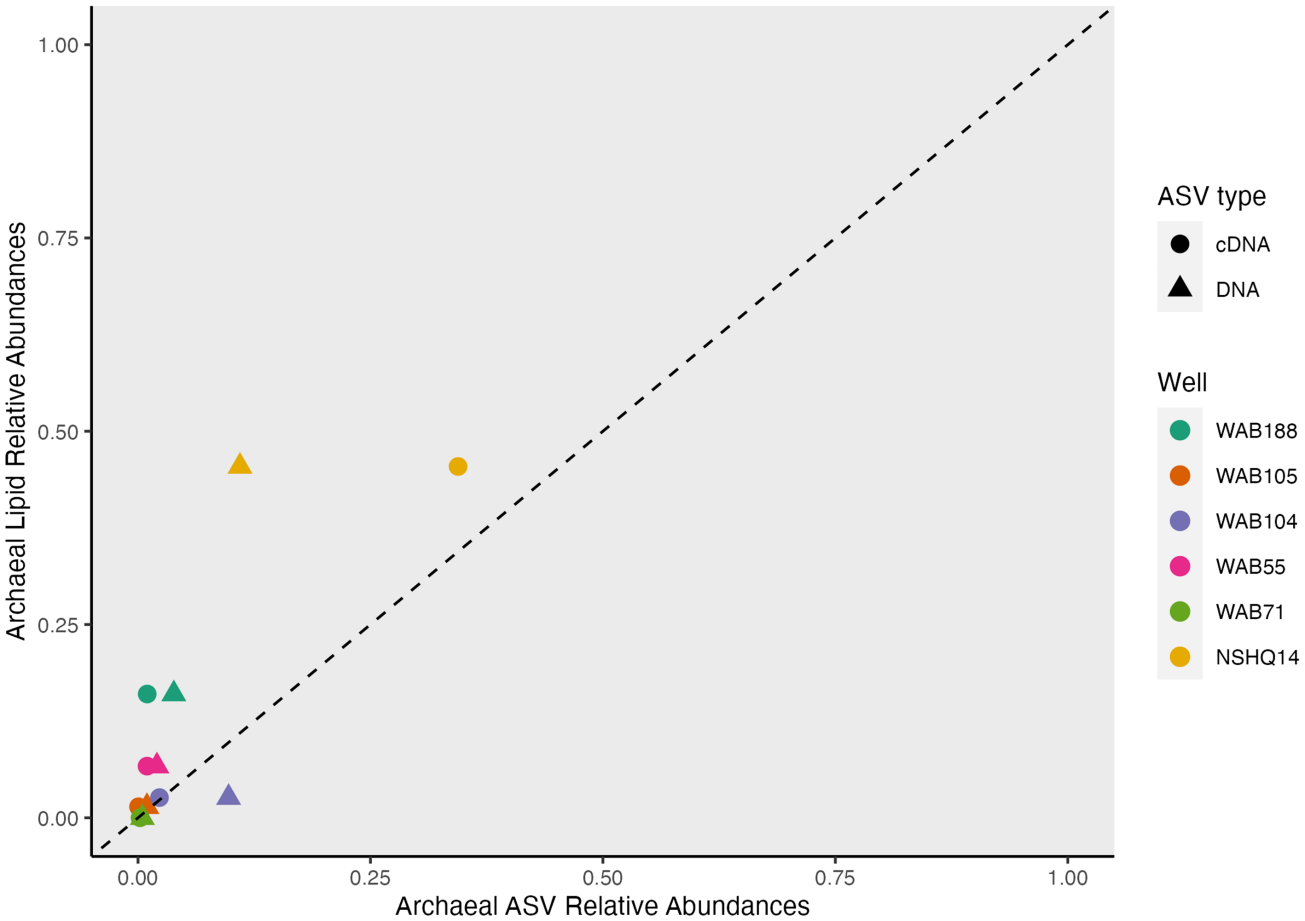
Overrepresentation of archaea in IPL compared to genomic and transcriptomic assays.

While archaeal lipids constituted >40% of the lipidome in all but one well, archaeal amplicon sequence variants (ASVs) comprised at most only 10.9% of the DNA and 34.4% of the cDNA (Figure 6). In wells WAB105 and WAB55, archaeal ASVs made up less than 2.5% of all ASVs; yet, 1G-AR accounted for 46 and 67% of the measured IPLs, respectively. However, fluids that exhibited the greatest relative abundance of lesser archaeal IPLs (e.g., 2G headgroups, GDGTs) did also have the greatest relative abundance of archaeal ASVs. Predominant archaeal ASVs varied considerably between wells; NSHQ14 and WAB188 were dominated by Methanobacteria, WAB55 and WAB104 by Nitrosopumiliaceae, and WAB105 by Woesarchaeales (Supplementary Table S5).

Pearson’s correlation was used to evaluate relationships between non-isoprenoidal IPL and bacterial ASV relative abundances. A total of 48 bacterial IPL compounds were correlated significantly (Bonferroni-corrected value of *p* < 0.005) with 84 bacterial ASVs (Supplementary Figure S3). Generally, most compounds within a lipid class correlated with the same ASV, but multiple ASVs demonstrated equivalent correlation coefficients and value of ps per lipid class. 1G and 2G ceramides correlated with an entirely different set of ASVs; 1G ceramides were associated with Acidobacteria, Ignavibacteria, Desulfomonile, Candidate Phylum DTB120, Nitrospinota, and Verrucomicrobiota, while 2G ceramides were associated primarily with Acetothermia and Meiothermus. Both glycosphingolipid classes correlated with different ASVs assigned to Firmicutes, Thermodesulfovibriona, Chloroflexi, Alphaproteobacteria, and Gammaproteobacteria. Trimethylated ornithine lipids were correlated with the same set of ASVs as 1G-ceramides, and NAcG-containing classes were correlated with the same set of ASVs as 2G-ceramides. Betaine lipids correlated with Gammaproteobacteria (primarily Pseudomonas), Pedosphaeraceae, Planctomycetiota, Nitrospirota, Firmicutes, Acidobacteria, and Bacteriodota (primarily Kryptioniales).

### 3.5. Trends in lipid composition with aqueous geochemistry

**Figure 7:**
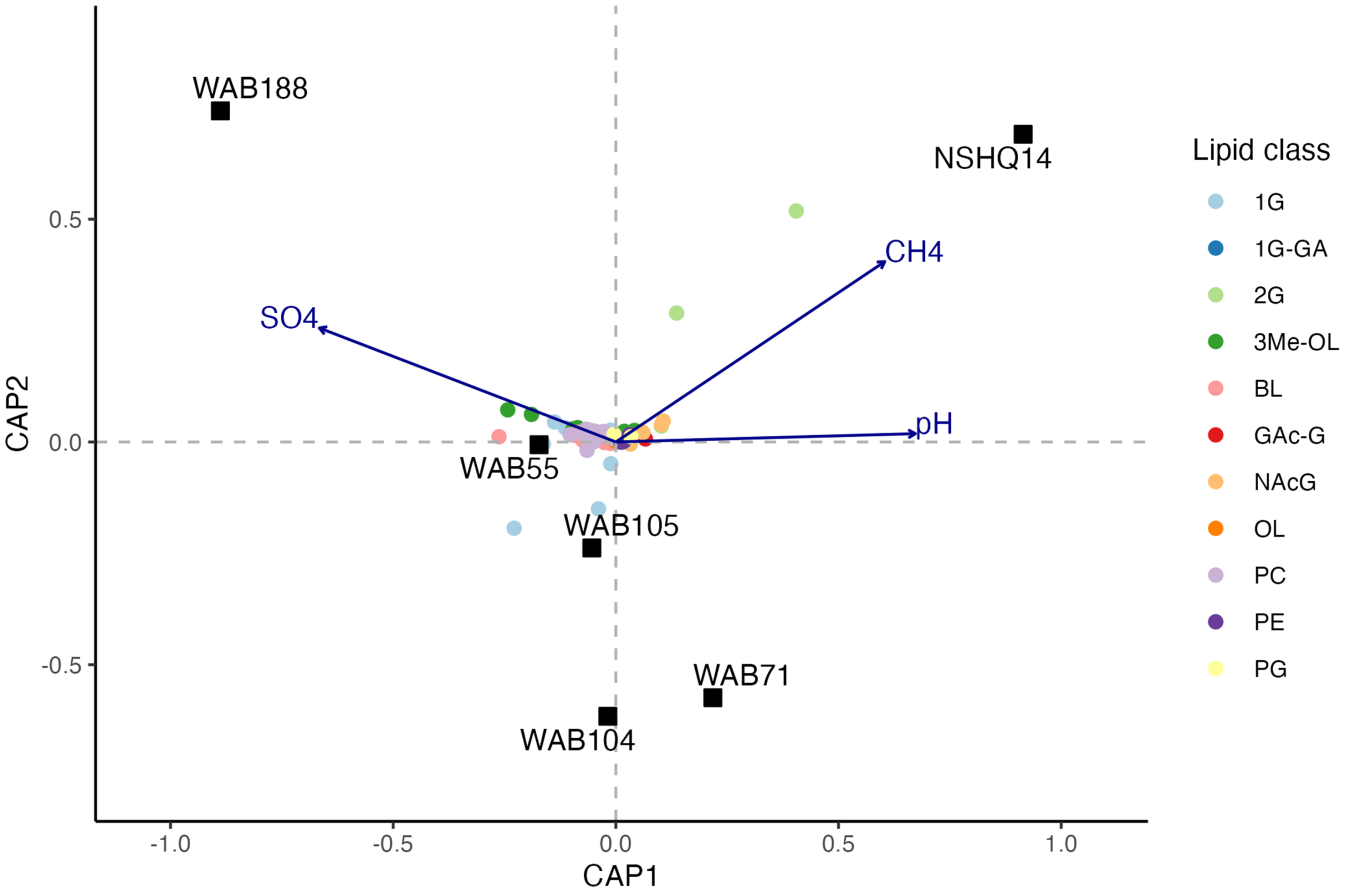
Constrained analysis of principle coordinates (CAP) of the Gower dissimilarity matrix of Hellinger-transformed IPL compound relative abundances. Permutations test of 500 iterations indicated significance of analysis (pseudo *F* = 2.33, value of *p* = 0.046).

To evaluate the relationship between the distribution of IPLs and subsurface fluid chemistry, we performed constrained analysis of principle coordinates (CAP) using the Gower dissimilarity matrix of IPL compound relative abundances for each site. For this analysis, we omitted 1G-AR from the calculation of IPL relative abundances, as this compound was strongly overrepresented in the intact polar lipidome compared to the relative abundance of any potential source organism as inferred by sequencing. Three explanatory variables (pH, [CH_4_], [SO_4_^2−^]) explained 77.7% of the observed variance (*R*^2^ = 0.77; 500 permutations: pseudo *F* = 2.33, value of *p* = 0.046) in the lipidome (Figure 7). Due to multicollinearity of geochemical parameters, replacing NO_3_^−^ for SO_4_^2−^ and H_2_ for CH_4_ yielded a similar ordination of samples and IPL compounds. The triplot for the CAP displayed a prevalence of trimethylated ornithine and betaine lipids in wells associated with higher concentrations of sulfate. Additionally, a trend toward 2G instead of 1G headgroups of glycolipids was demonstrated in wells with higher methane concentrations and more hyperalkaline pH.

**Figure 8:**
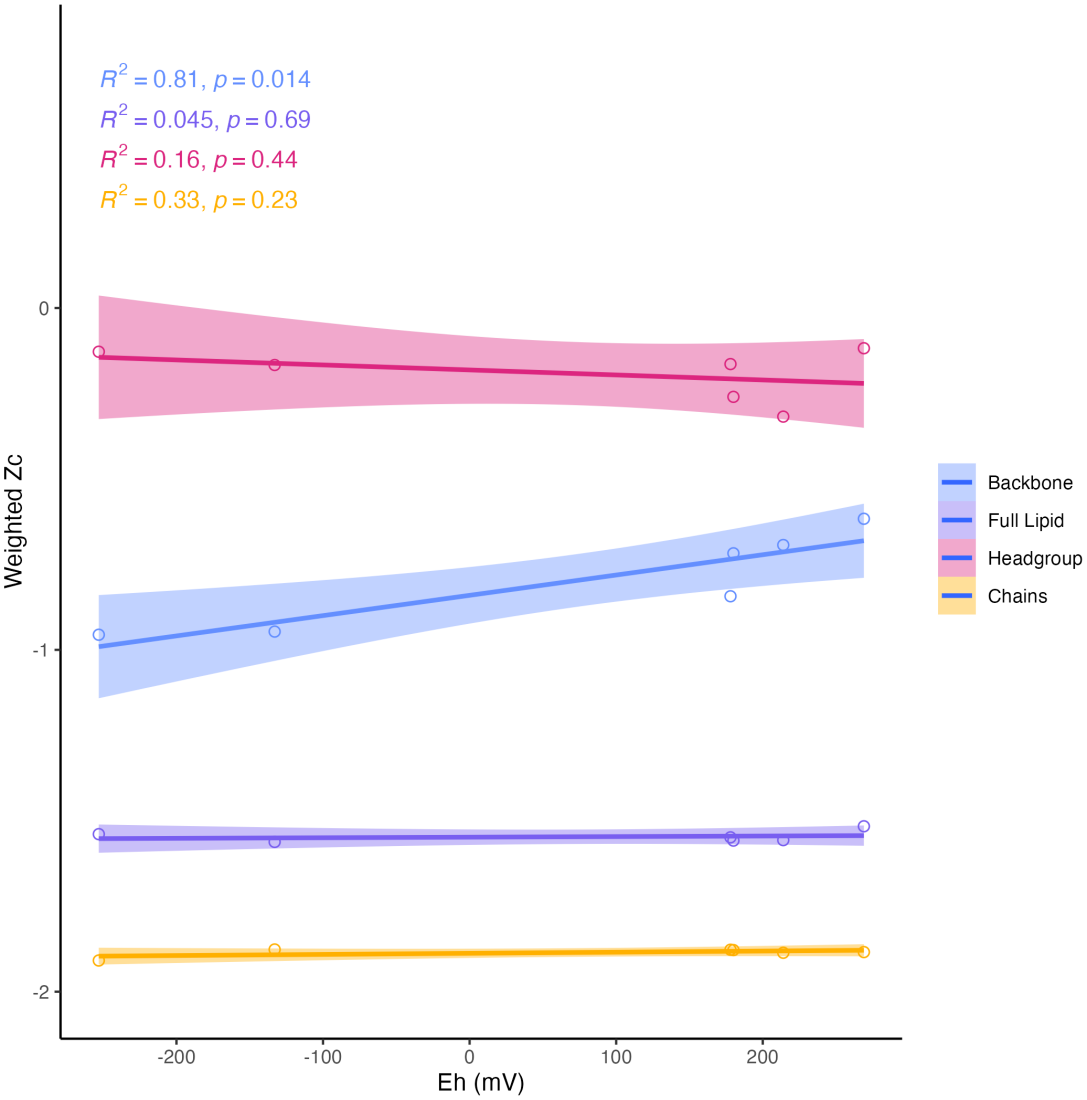
Abundance-weighted carbon oxidation state (Zc) of intact lipids, headgroups, backbones, and chains in relation to Eh. Backbone Zc is significantly (R^2^ = 0.81, *p* = 0.014) correlated with Eh (mV) of fluids.

Differences in overall lipid structure across wells were also assessed through calculation of the average oxidation state of carbon (Z_c_) in IPLs. Lower values of Z_c_ in a molecule indicate more reduced carbon (e.g., −4 in CH_4_) and higher values represent more oxidized carbon (e.g., +4 in CO_2_). Across sampled fluids, the abundance-weighted Z_c_ of the full lipid and of the lipid chains were remarkably consistent, with the full lipid Z_c_ varying from −1.52 to −1.56 and the chain Z_c_ from −1.88 to −1.90. The Z_c_ of headgroups and backbones were slightly more variable across samples, ranging from −0.12 to −0.32 and from −0.62 to −0.96, respectively (Supplementary Table S6). Notably, all calculated components of the IPL exhibited negative carbon oxidation states. The abundance-weighted Z_c_ of IPL backbones was the only component of the IPL to display any significant trend with geochemistry (Figure 8), which demonstrated a positive correlation with Eh (*R*^2^ = 0.81, *p* = 0.014).

The proportion of ether-linked chains was greatest in hyperalkaline wells NSHQ14 and WAB71, where negative Eh values were measured (Supplementary Figure S4). Ester linkages were nearly absent in both these wells, but were the second most common linkage in all other sampled wells. Amide linkages were most abundant in well WAB55 (7% of linkages). There was little variation in both the abundance-weighted average number of aliphatic carbon (nC: 16.5–17.6) and unsaturations (nUns: 0.3–0.51) in lipid chains across sampled fluids. The maximum nC and minimum nUns for lipid chains was reported in NSHQ14, the well with the most negative Eh, however, there was no statistically significant trend of chain-linkage, nC, or nUns with Eh or any other measured geochemical parameter.

## 4. Discussion

### 4.1. Subsurface intact polar lipidome of a terrestrial serpentinite

Archaeal IPLs in the Samail Ophiolite were dominated by those with an archaeol core. Diether lipids are not typically found as the dominant class of archaeal lipids in ecosystems (Koga and Morii, 2005; Biddle et al., 2006; Sollai et al., 2019). However, a predominance of diether over tetraether IPLs has been described at a marine serpentinizing system- the Lost City hydrothermal vent field (Bradley et al., 2009b; Lincoln et al., 2013), in ophiolites (Rattray et al., 2022), as well as in deep oceanic crust drilled from the Atlantis Bank (Li et al., 2020) and some sites of methane seeps (Rossel et al., 2011). It has been postulated that archaeol abundance may serve as an indicator for methanogenic biomass, particularly where the Thaumarchaeotal GDGT lipid crenarchaeol (GDGT-5) is low in abundance (Lim et al., 2012). We found 2G-AR and 2G-GDGT-0 to be most abundant in the well NSHQ14 where methane concentrations were highest, ASVs assigned to *Methanobacterium* were the most prominent, and cDNA for this organism was comparatively enriched (Figures 2–4). In culture, isolates of *Methanobacterium* have been reported to produce both archaeol and GDGT-0 with glycosyl and phosphate-containing headgroups (Nishihara and Koga, 1987). While we did detect Thaumarcheota of the family Nitrososphaeraceae (Supplementary Table S5), especially in alkaline peridotite-hosted fluids, we did not detect crenarchaeol, the major lipid constituent of this family (Elling et al., 2017). It is possible intact crenarchaeol lipids are present at low abundances in sampled fluids, but not in high enough concentrations to be detected by MS2 analysis. Accordingly, it is possible that some small fraction of isoprenoidal IPLs, including a proportion of 1G-AR, could be produced by Nitrososphaeraceae and other archaeal taxa, but these organisms are unlikely to be the major contributor of isoprenoidal diether lipids (Pitcher et al., 2010). We instead suggest the abundance of archaeol-based lipids in these fluids likely represent primarily a methanogenic source. The idea that at least some fraction of the archaeal IPL pool is actively produced by methanogens can be supported by previous studies of the biomass in these fluids by Fones et al. (2019) showing active biological ^14^CH_4_ production from ^14^C-labeled bicarbonate and by Kraus et al. (2021) identifying transcripts for key genes encoding methanogenesis enzymes.

Glycolipids with non-isoprenoidal diether cores were ubiquitous in biomass sampled from the Samail serpentinite-hosted fluids. Bacterial diether glycolipids were the major lipid class detected at the Lost City Hydrothermal Field (Bradley et al., 2009a,b). Non-isoprenoidal diether core lipids have additionally been described in fossilized serpentinite systems in the Iberian Margin (Klein et al., 2015), in serpentinite rock in ophiolites (Zwicker et al., 2018; Newman et al., 2020), and in drill cores of deep oceanic crust at Atlantis Bank (Li et al., 2020), suggesting this lipid class is common across ecosystems supported by water-rock interaction. Only a limited number of cultured bacteria have been found to synthesize DEG backbones, stemming mostly from thermophilic clades such as *Thermodesulfobacterium* (Langworthy et al., 1983), *Ammonifex* (Huber et al., 1996), *Aquifex* (Huber et al., 1992), *Rhodothermus* (Jorge et al., 2015), and *Thermatoga* (Damsté et al., 2007), along with some mesophilic sulfate-reducers (Rütters et al., 2001; Grossi et al., 2015). The only cultured isolate that has been reported to produce DEG lipids with a glycosyl headgroup is *Thermodesulfobacterium* (Langworthy et al., 1983). We observed the greatest relative abundance of 1G-DEG lipids in well WAB71 where the most predominant ASV was assigned to the family Thermodesulfovibriona (Figure 5), supporting the hypothesis put forth by Bradley et al. (2009a) that sulfate-reducing organisms could be the source of these enigmatic lipids in serpentinized fluids (Bradley et al., 2009a). However, at Lost City, no known strains of bacteria capable of producing DEG lipids were detected via 16S rRNA gene sequencing, and so it was postulated that Clostridial sulfate-reducers instead could be a possible source (Bradley et al., 2009a). We did observe Clostridial ASVs enriched in the cDNA fraction of WAB71 as well as minor relative abundances of ASVs assigned to Aquificales, Ammonifexales (e.g., *Desulforudis*), and Desulfobacteriota, so other potential source organisms cannot be ruled out.

Although diether glycolipids were measured in high abundance across all measured samples, it is important to note that there is no apparent instrumental bias toward the detection of this class of lipids. Instead, the ionization response of both mono- and di-glycosyl standards was an order of magnitude lower (1.84E+05 and 1.21E+06, respectively) than that of aminolipids (2.37E+08) and most phospholipids (1.81E+06 to 1.59E+08; Supplementary Table S3, Supplementary Figure S1) with no observed effect on ionization response for ether compared to ester linkages to the glycerol backbone (e.g., PC-AR and PC-DAG 32:0 standards: 8.85E+07 and 7.04E+07, respectively).

Minor bacterial IPLs in the lipidome included betaine and ornithine (including trimethylated ornithine) aminolipids, glycosphingolipids, aminoglycolipids with a N-acetyl glucosaminyl headgroup (NAcG), and to a lesser degree, phospholipids. We used Pearson’s correlations between the relative abundances of non-isoprenoidal lipids and bacterial ASVs to discern possible sources of these largely cosmopolitan IPLs (Supplementary Figure S3). The significant correlation of highly specific lipids with NAcG headgroups to *Meiothermus* ASVs lends credence to the use of this approach because this class of lipids has only been found in members of the Thermus/Meiothermus clade (Ferreira et al., 1999; Yang et al., 2006). However, 14 other ASVs also significantly correlated with NAcG structures (Supplementary Figure S3). Because microbial community composition in Samail Ophiolite fluids is associated with aqueous geochemistry (Rempfert et al., 2017), it is to be expected that the relative abundance of many taxa co-vary, thus complicating source assignment.

Ceramide-linked sphingolipids were common across sampled fluids. Ceramide backbone linkages are only known to be synthesized by a few bacterial groups. Sphingolipids have been reported in organisms belonging to the Fibrobacteres-Chlorobi-Bacteriodetes superphylum, Alphaproteobacteria (e.g., *Sphingomonadales*), and some Deltaproteobacteria (Olsen and Jantzen, 2001; Keck et al., 2011; Stankeviciute et al., 2022), however, the genetic biosynthetic potential is found in a wider range of Gram-negative and several Gram-positive genera including *Thermodesulfovibrio* and *Ignavibacter* (Sohlenkamp and Geiger, 2016; Stankeviciute et al., 2022). 1G sphingolipids were correlated with Bacteriodota, Ignavibacteriales, and Thermodesulfovibriona ASVs, and both 1G and 2G glycosphingolipids were significantly correlated with ASVs belonging to Alpha- and Gammaproteobacteria (Supplementary Figure S3); we tentatively attribute ceramide IPLs to a combination of these taxa. While ceramide lipids are much more common in Eukarya, Kraus et al. (2021) reported only low eukaryotic 18S rRNA gene sequence counts in complimentary samples (0.11% of all sequences from all wells), and so we assume a bacterial source.

Aminolipids, such as betaine and ornithine lipids, were relatively abundant to the greatest degree in alkaline wells and were associated with higher concentrations of oxidants (e.g., sulfate) in constrained principle coordinate analysis (Figure 7). To the best of our knowledge, betaine lipids have only been reported in Alphaproteobacteria, Gammaproteobacteria, Actinobacteria, Verrucomicrobia, and Bacteriodetes (Benning et al., 1995; Geiger et al., 1999; Geske et al., 2013; Sebastián et al., 2016; Yao et al., 2016). Betaine lipids with DGTS/DGTA headgroups were significantly correlated with all these clades (Supplementary Figure S3), and thus we speculate this IPL class is produced by multiple bacterial sources in this setting. DGTS and DGTA have identical fragmentation patterns in MS2, but we presume these aminolipids have DGTS headgroups because DGTA has not yet been identified in bacteria (Geiger et al., 2010; López-Lara and Geiger, 2017). Ornithine lipids are only present in Bacteria (Vences-Guzmán et al., 2012) and have been widely reported in Proteobacteria and other Gram-negative bacterial strains (Geiger et al., 2010; Sohlenkamp and Geiger, 2016). Like betaine lipids, ornithine lipids are likely produced by many taxa in this environment. Interestingly, while Planctomycetes groups were detected in low relative abundances in alkaline wells, there was no significant correlation of these groups with the abundance of trimethylated ornithine lipids (3Me-OL). Planctomycetes is the only known source of 3Me-OL signatures (Moore et al., 2013; Moore, 2021). However, 3Me-OL IPLs did correlate with Verrucromicrobia ASV abundance, a phylum that also belongs to the PVC superphylum (Planctomycetes-Verrucomicrobia-Chlamydiae). It is thus possible that the ability to produce this trimethylated structure is more widely spread across phyla than previously described.

### 4.2. Membrane adaptations in a polyextreme environment

The abundance of glycolipids and aminolipids compared to phospholipids in serpentinized fluids resembles the headgroup assemblages typically observed in the oligotrophic open ocean where phosphate is limiting (Van Mooy et al., 2009; Schubotz et al., 2018; Schubotz, 2019). All wells sampled did not have any detectable phosphate (detection limit 5 μM), which indicates subsurface fluid phosphate concentrations are below the 10 μM concentration at which heterotrophic (Sebastián et al., 2016) and sulfate-reducing bacteria (Bosak et al., 2016), as well as methanogenic archaea (Yoshinaga et al., 2015), have been observed to replace phospholipid membrane lipids with amino- and/or glycolipids in culture. Peridotite rocks have exceedingly low bulk phosphorus concentrations (median of 131 ppm from 577 samples in the EarthChem database; Porder and Ramachandran, 2013), with harzburgites in the Samail Ophiolite containing only 0.009–0.012 wt.% P_2_O_5_ (Hanghøj et al., 2010). Thus, limited phosphorus is available in the host rock to be liberated as phosphate during water-rock reaction. Additionally, the limited availability of phosphate in subsurface fluids within the ophiolite may be due to the formation of insoluble Ca-phosphates in hyperalkaline Ca-OH fluids, as well as the presence of the mineral brucite, which was found to comprise up to 8 wt% of the mineral assemblage in Samail Ophiolite dunite (Templeton et al., 2021) and is known to be an effective scavenger of phosphate from fluids (Holm et al., 2006; Templeton and Ellison, 2020). Accordingly, we suggest that the predominance of glycolipids and aminolipids in serpentinized fluids in Oman represents a phosphorus conservation strategy of organisms adapted to living within serpentinite systems with notably low phosphate availability.

Through culturing experiments, it has been documented that anionic phospholipids (e.g., PG) are often replaced with anionic glycolipids (e.g., GAc), and that neutral or zwitterionic phospholipids (e.g., PE, PC, PME, PDME) are replaced with neutral or zwitterionic amino or glycolipids (e.g., 1G, 2G, DGTS, OL) during phosphate limitation (Geske et al., 2013; Carini et al., 2015; Bosak et al., 2016; Sebastián et al., 2016). However, the ratio of anionic to neutral or zwitterionic lipids is not always conserved through membrane remodeling, which may impact the integrity of the lipid membrane (Bosak et al., 2016; Schubotz, 2019). Additionally, the shape of the membrane may be altered during remodeling (Bosak et al., 2016) if the configuration of cylindrical bilayer-forming IPLs and conical non-bilayer-forming IPLs is adjusted (Schubotz, 2019). For organisms that cannot synthesize DGTS, zwitterionic ornithine lipids have been implicated as important for maintaining membrane lipid charge (López-Lara et al., 2005). The addition of three methyl groups on the terminal nitrogen of trimethylated ornithine lipids mimics the structure of a phosphocholine headgroup, which likely imparts a greater polarity and similar cylindrical shape (Moore et al., 2013; Moore, 2021). Phosphocholine is a bilayer-stabilizing IPL common in heat-stressed microorganisms (Hazel and Williams, 1990; Hazel, 1995; Sollich et al., 2017) and was the most abundant phospholipid in sampled fluids. Accordingly, the predominance of zwitterionic 3Me-OL and DGTS aminolipids in the Samail Ophiolite lipidome likely reflect a preservation of membrane charge, structure, and integrity through membrane adaptation.

In addition to the apparent adaptation of IPL headgroup composition to phosphate limitation in serpentinite-hosted fluids, we observed an influence of fluid geochemistry on IPL backbone linkage structures. The average carbon oxidation state (Z_c_) of IPL backbones was significantly and positively correlated with the measured reduction potential (Eh) of sampled fluids (Figure 8). Fones et al. (2019) noted a similar trend with the proteome’s Z_c_ in complimentary samples, with the lowest Z_c_ observed in the most hyperalkaline, reduced well, NSHQ14. It has been interpreted that this relationship reflects an evolutionary convergence to minimize cellular biosynthetic costs, as reduced biomolecules are energetically more cost-effective to synthesize under reducing conditions (Dick and Shock, 2011; Dick, 2014; Fones et al., 2019; Boyer et al., 2020). Boyer et al. (2020) thus postulated that the Z_c_ of lipids represents an adaptation of organisms to the availability of reduction potential (i.e., concentrations of electron donors and acceptors) in their environment. However, we did not observe any significant correlation of full lipid Z_c_ with Eh, as was reported for hot spring IPL samples from Yellowstone (Boyer et al., 2020). The invariance of IPL Z_c_ in Samail Ophiolite aquifers can likely be attributed to the pervasiveness of phosphate limitation. Due to the weight of nitrogen and phosphorus in the calculation of lipid Z_c_, the ubiquity of headgroup modifications across sampled fluids overprints the observed signature from backbone modifications.

The reduced IPL-backbone Z_c_ in NSHQ14 corresponds to a prevalence of ether linkages, the most reduced backbone configuration explored in this study (Figure 1). Ether-linked backbones have commonly been described in deep marine sediments (Evans et al., 2017), hydrothermal ecosystems (Jahnke et al., 2001; Pancost et al., 2006), and anerobic methane oxidizing and sulfate-reducing consortia at cold methane seeps (Hinrichs et al., 2000; Orphan et al., 2001; Pancost et al., 2001; Rossel et al., 2011) where their abundance was attributed to the robustness of these lipid structures. However, it is also possible ether linkages were prevalent in these environments in part due to the energetic favorability of producing this backbone structure under the prevailing reducing conditions. Ether-bound lipids have a lower proton permeability relative to ester-bound lipids (van de Vossenberg et al., 1998; Mathai et al., 2001) which may reduce energetic costs to cellular maintenance in energy-limiting environments (Valentine, 2007). Ether-bound lipids are also produced by soil bacteria during starvation-induced sporulation (Ring et al., 2006; Lorenzen et al., 2014), further implicating an influence of energy availability on membrane lipid backbone structure in serpentinizing settings where oxidants and inorganic carbon are deficient.

Ceramide backbone linkages also correspond to a reduced Z_c_ (−0.75) and were observed in the lipidome of all sampled fluids (Supplementary Figure S4). Ceramides have been reported as a major component of the lipidome in hyperthermic marine sediments in Spathi Bay (Sollich et al., 2017), as well as minor components in serpentine rock in the Chimera ophiolite (Rattray et al., 2022), the anoxic water column of the Black Sea (Schubotz et al., 2009), and in hot springs (Schubotz et al., 2013). However, ceramides are more commonly described for their hypothesized role in virulence and stress survival in host-associated taxa (e.g., Kunz and Kozjak-Pavlovic, 2019). Sollich et al. (2017) hypothesized that ceramides may confer increased membrane rigidity and stability through tighter membrane packing facilitated by the strong hydrogen-bonding potential of ceramide backbones. This hypothesis is supported by multiple studies that confirm intramolecular hydrogen bonding of amino and hydroxyl groups in the ceramide backbone with the headgroup of sphingomyelin lipids (Talbott et al., 2000; Mombelli et al., 2003; Venable et al., 2014). Interestingly, the availability of an amino group near the lipid headgroup also occurs in the fatty amide backbone structure of ornithine lipids, implying similar potential for increased hydrogen bonding. Overall, ceramide lipids likely provide increased membrane stability in IPLs and perhaps represent an alternative to ether linkages for organisms not genetically capable of synthesizing DEG backbones.

We observed little variation in the abundance-weighted average number of aliphatic carbons and unsaturations in IPL chains across wells; however, we did note the maximum nC and the minimum nUns in the chains of IPLs from the well NSHQ14 where pH was highest. Increasing length and saturation of alkyl chains has been shown to decrease membrane permeability (de Gier et al., 1968; Paula et al., 1996) which could modulate the maintenance of an ion gradient across the membrane at high pH. The methods employed for IPL characterization do not allow us to distinguish between branched non-isoprenoidal chains (e.g., iso- and anteiso- fatty acids) and straight-chain counterparts, and so nC is not necessarily equivalent to chain length. However, increased branching of aliphatic chains, which would similarly increase nC, has been reported in laboratory cultures of organisms belonging to the Bacillus/Clostridium subphylum when grown at high pH (Clejan et al., 1986; Li et al., 1994; Prowe and Antranikian, 2001). Thus, the observed trend of increasing nC and decreasing nUns could represent a strategy to cope with hyperalkaline pH. Importantly, unlike headgroup modifications, adjustments to IPL chains such as increased methylations or chain length could ultimately be preserved in fossil biomarker structures such as hydrocarbons as a record of past geochemical conditions.

### 4.3. IPLs as biomarkers for living biomass in terrestrial serpentinizing environments

A key assumption in the use of IPLs to characterize the abundance and physiology of microorganisms is that IPLs are a proxy for living biomass. This has been widely assumed because the bond linking the polar headgroup to the lipid backbone is labile and can easily be cleaved after cell death on the order of hours to days (White et al., 1979; Harvey et al., 1986). However, the lack of a meaningful correlation of estimated IPL concentrations with enumerated planktonic cells in Samail Ophiolite subsurface fluids (Supplementary Figure S2) demonstrates a stark difference in relative turnover times between at least some fraction of IPLs and microbial communities. This, in conjunction with the predominance of 1G-AR in wells that exhibited very low relative abundances of archaeal DNA and RNA (Figure 6) suggests an unknown, but large, proportion of IPLs in this setting does not represent viable biomass. An overrepresentation of archaea by IPL analyses in comparison to other biomass quantification techniques has been reported in multiple marine deep biosphere surveys (Biddle et al., 2006; Lipp et al., 2008; Lengger et al., 2014). The prevalence of archaeal IPLs in deep, subsurface environments has been interpreted to be an artifact of the differential lability of ester-bound bacterial and ether-bound archaeal IPLs (Schouten et al., 2010). Laboratory studies of IPL degradation kinetics demonstrate that glycosidic archaeal IPLs degrade at a rate of one to two orders of magnitude slower than bacterial phosphatidic ester-bound IPLs (Logemann et al., 2011; Xie et al., 2013). In deep biosphere systems with very low cellular turnover rates, it has been extrapolated that glycosidic archaeal lipids could persist for 10 of 1,000 of years (Lin et al., 2013; Xie et al., 2013). Because of the persistence of at least some classes of IPLs in serpentinite-hosted fluids, the intact polar lipidome must be considered as a cumulative record of longer-scale patterns of microbial diversity and geochemistry, and not as a snapshot of a dynamic system.

### 4.4. Lipid preservation potential

The persistence of IPLs in fluids beyond the presence of their source organisms is promising for the detection of extinct life through lipid biomarker signatures. Intact diether lipids were found to be highly abundant in hyperalkaline fluids (Figure 3) despite low planktonic cell abundances, indicating a potential for these biomolecules to accumulate in fluids. Over time, these IPLs could amass to concentrations far surpassing living biomass, thus facilitating the detection of lipid signatures if these biomarkers were to be preserved in mineral precipitants. Fossil lipid signatures have been observed in carbonate and brucite veins at both terrestrial and marine sites of serpentinization (Klein et al., 2015; Zwicker et al., 2018; Newman et al., 2020), supporting the hypothesis that fluid-sourced microbial membrane lipids can be preserved in this type of geologic setting. Although, the apparent difference in residence times between diether lipids and other IPL classes suggests the preserved lipid biomarker record would not be a representative snapshot of the full diversity of the microbial community. Nevertheless, the detectability of IPLs (3.3 ng/L – 27 mg/L) in fluids with low (~1 × 10^5^ cells/mL) cellular abundances merits further investigation into serpentinites as targets for life detection on other planetary bodies. Specifically, it supports the concept that life detection could be achieved via analysis of organic molecules in serpentinite fluids or in secondary mineral assemblages, to search for degradation products of cell membranes.

### 4.5. Reinterpretation of the fossil lipid biomarker record of the Samail ophiolite

The intact polar lipidome of serpentinized fluids hosted within the Samail Ophiolite can provide valuable context to aid in the reconstruction of past microbial activity from core lipid records of terrestrial serpentinites. Newman et al. (2020) surveyed core lipid biomarkers in travertine deposits and carbonate veins of serpentinized rock in the Samail Ophiolite. This record represents an integrated signature of microbial life over the time at which carbonate was precipitated, which could span ~50,000 years (Clark and Fontes, 1990; Kelemen and Matter, 2008; Kelemen et al., 2011; Mervine et al., 2014). The core structures observed here in IPLs from subsurface Samail Ophiolite fluids most closely resemble the assemblage of lipids detected in layered carbonate and travertine outcrops (Subset A in Newman et al., 2020), which were categorized by an abundance of archaeol and non-isoprenoidal ether lipids as well as a high ratio of GDGT-0 to crenarchaeol. These lipids were interpreted as signatures of surficial microbial communities because the source carbonates were presumed to have precipitated from surface outflow of Ca^2+^ and OH^−^ rich, serpentinized fluids (Newman et al., 2020). We posit that microorganisms inhabiting deep, highly reacted fluids could alternatively be the source of these biomarkers.

In wells such as NSHQ14, we sampled highly reacted fluids that exhibited a hyperalkaline pH (11.3) with reduced Eh (−253 mV), as well as characteristically high calcium (4,340 μM) and low magnesium (5 μM) and DIC (130 μM) concentrations consistent with the expected chemistry for source fluids of travertines in serpentinizing settings (Barnes and O’neil, 1969; Neal and Stanger, 1985; Paukert et al., 2012). Highly reduced fluids contained co-occurring intact diglycosyl archaeol and GDGT-0 lipids with non-isoprenoidal glycosyl diether bacterial lipids which were associated with high relative abundances of cDNA of methanogenic *Methanobacteria* and sulfate-reducing Thermodesulfovibriona. Because we found archaeal diether IPLs to be so recalcitrant in serpentinized fluids, we hypothesize archaeol, and perhaps other ether-linked lipids, comparatively accumulate in subsurface fluids with respect to more labile lipids, and thus are preferentially preserved upon rapid carbonate precipitation as reacted, subsurface fluids reemerge near the surface and are exposed to atmospheric carbon dioxide. We also note that the abundance of GDGT-0 could primarily be sourced by *Methanobacteria*, and not methanotrophic archaea, Crenarcheota, or Thaumarcheota as suggested by Newman et al. (2020), because we observed such high relative abundances of 2G-GDGT-0 in association with *Methanobacteria*. A subsurface, methanogenic source of archaeal biomarkers in serpentinites is consistent with the findings by Zwicker et al. (2018) for the serpentinite-hosted Chimera seeps of Turkey, as well as the distribution of methanogenic biomarkers in marine serpentinites where archaeol and acyclic GDGTs were most abundant within chimneys (Bradley et al., 2009a; Lincoln et al., 2013) and subsurface veins (Klein et al., 2015). The co-occurrence of bacterial non-isoprenoidal ether-linked lipids with archaeol in all intact and fossil lipid surveys of alkaline sites of serpentinization suggests these biomarkers could be a hallmark signature of serpentinized fluids (Bradley et al., 2009a,b; Lincoln et al., 2013; Méhay et al., 2013; Klein et al., 2015; Zwicker et al., 2018; Newman et al., 2020).

### 4.6. Implications for the detection of biomarkers on Mars

The detection of serpentine minerals by the Compact Reconnaissance Imaging Spectrometer for Mars (CRISM) in conjunction with olivine-rich basalts, carbonates, and other alteration mineral phases (e.g., talc, saponite) indicates serpentinization on Mars was once active, and potentially widespread (Ehlmann et al., 2008, 2009, 2010; Brown et al., 2010). Spectroscopic evidence from the Perseverance rover for aqueously-altered, olivine-rich rocks (Farley et al., 2022) imply there may even be the potential for sample collection of serpentinized Martian rock in the near future. We suggest these serpentine mineral assemblages are promising potential targets in the search for preserved organic signatures of ancient biomass on Mars. The abundance of recalcitrant IPLs in subsurface serpentinite-hosted fluids reported in this study in combination with the detection of fossil signatures in Samail Ophiolite travertines reported by Newman et al. (2020) support the theory that subsurface microbial communities inhabiting serpentinite-hosted aquifers could be preserved within mineral products of fluid-rock reaction. Travertine deposits form where deep-seated, highly reacted fluids discharge at the surface from bedrock fissures (Giampouras et al., 2020). Thus, travertine deposits facilitate the accessibility of sampling fossil subsurface, fluid-hosted biomass. On Mars, faults from impacts, the buildup of Tharsis, the dichotomy-forming event, or local tectonics (e.g., subsidence, uplift) could penetrate deep into the subsurface and potentially act as conduits for fluid seepage (Oehler and Etiope, 2017), thus providing near-surface access to subsurface material. A subsurface source of biomass may promote its preservation in carbonate veins below the surface, as a major challenge for the persistence of organic signatures on the Martian surface is degradation from exposure to ionizing radiation or prevalent chemical oxidants such as perchlorates (Hays et al., 2017). Accordingly, locations such as Nilli Fossae where fractured, serpentinized rocks have been detected from orbit should be prioritized for detection of preserved cell-derived organics in future missions.

## 5. Conclusion

This study represents the first intact polar lipid biomarker survey of subsurface fluids from a terrestrial site of active serpentinization, sampling high pH fluids characterized by challenging states of nutrient and energy limitation, thus greatly increasing the astrobiologically relevant physicochemical conditions explored in lipid biomarker investigations. To probe the anticipated diversity of intact lipid structures in this setting, IPLs were inventoried using an expansive, custom environmental lipid database, which expands the application of targeted and untargeted lipidomics in the study of microbial and biogeochemical processes. The intact polar lipidome across fluids was dominated by archaeal and bacterial glycosyl diether lipids that bore a surprising resemblance to the biomarker assemblages described for the marine serpentinizing system, Lost City Hydrothermal Field (Bradley et al., 2009a), despite differing microbial community compositions. In Oman, we interpret these lipids to most likely be signatures of methanogenic archaea belonging to the genus *Methanobacteria* and sulfate-reducing bacteria, possibly of the family Thermodesulfovibriona or Clostridiaceae. The co-occurrence of bacterial and archaeal diether glycolipids at Lost City and in the Samail Ophiolite suggests these biomarkers are potentially diagnostic signatures for serpentinizing systems.

The prominence of non-phospholipids such as betaine, trimethyl-ornithine, and glycosphingolipids suggests extensive membrane modifications of polar headgroups, likely as a conservation strategy of organisms adapted to living in phosphate-depleted conditions within serpentinite rock and fluids. Additionally, backbone linkages in highly reduced fluids exhibited low carbon oxidation states and were characterized by primarily ether and amide linkages, which may reflect an energy-conservation strategy of cells and an adaptation to reduce membrane permeability.

An unknown, but possibly dominant, proportion of IPLs in this setting were not representative of living biomass as commonly assumed for intact lipids. The accumulation of recalcitrant IPLs could facilitate the detectability of lipid biosignatures in mineral precipitates of serpentinized fluids, thus improving prospects for life detection efforts in serpentinites. We invoke this mechanism in a reinterpretation of the core lipid biomarker record for travertines in the Samail Ophiolite published by Newman et al. (2020), and hypothesize these biomarkers are representative of deep, highly reacted fluids rather than surficial microbial communities. This hypothesis merits further investigation because it implies that travertines should be a high priority target for organic biosignature detection on Mars or other planetary bodies. Overall, this work provides context for the interpretation of molecular fossil records in serpentinite-hosted settings, such as those potentially left on early Earth, Mars, or similar planetary systems, which will help guide future efforts to detect signatures of subsurface life.

## Data availability statement

The datasets presented in this study can be found in online repositories. Raw mass spectral data are deposited in the EBI MetaboLights repository under accession number MTBLS7570: https://www.ebi.ac.uk/metabolights/MTBLS7570. Raw sequences are deposited in the NCBI Sequence Read Archive (SRA) under accession number PRJNA560313. All data and source code used to produce the figures and data tables in this manuscript are available at https://github.com/krempfert/Samail_fluid_IPLs.

## Author contributions

AT, KR, and JuS conceived the study. KR, DN, EK, JoS, and AT collected the samples in the field. KR, DN, EK, ND, and JuS analyzed the samples. KR produced the expansive custom environmental lipid database, processed data, performed statistical analyses, and wrote the manuscript. All authors interpreted data and critically revised the manuscript text and figures. All authors contributed to the article and approved the submitted version.

## Footnotes

^1^ https://www.genome.duke.edu

^2^ https://github.com/Zymo-Research/figaro

^3^ https://github.com/danote/Samail_16S_compilation

^4^ https://github.com/krempfert/Samail_fluid_IPLs

^5^ https://github.com/krempfert/Samail_fluid_IPLs

^6^ https://gitlab.com/gmboyer/polarlipidzc

^7^ https://github.com/AJProbst/lip_metgen
