# Supplementary material for: Intact polar lipidome and membrane adaptations of microbial communities inhabiting serpentinite-hosted fluids
Source: Front Microbiol. 2023 Nov 10;14:1198786. doi: 10.3389/fmicb.2023.1198786 (PMC10667739; doi:10.3389/fmicb.2023.1198786)
Supplement: Supplementary file 7 [file Data_Sheet_1.docx]

Supplementary Material

Intact Polar Lipidome and Membrane Adaptations of Microbial Communities Inhabiting Serpentinite-Hosted Fluids

Kaitlin R. Rempfert^*^, Emily A. Kraus, Daniel B. Nothaft, Nadia Dildar, John R. Spear, Julio Sepúlveda, Alexis S. Templeton^*^

*** Correspondence:** Kaitlin Rempfert: [kaitlin.rempfert@colorado.edu](mailto:kaitlin.rempfert@colorado.edu)

Alexis Templeton: [alexis.templeton@colorado.edu](mailto:alexis.templeton@colorado.edu)

# Supplementary Text

## Assumptions for DEG backbone assignments

The abundance of bacterial diether glycolipids may be overestimated if peaks attributed to 1G-DEG lipids corresponded to 1G-1,2-alkanediols in some samples. These two compounds cannot be distinguished by mass or retention time because unsaturated IPLs with DEG backbones have identical chemical formulas to saturated 1,2 alkanediols with one less carbon in the core structure (e.g. the chemical formula for both 1G-DEG 32:1 and 1G-alkanediol 31:0 is C_41_H_80_O_8_) and coelute due to having the same headgroup composition. Negatively ionized MS2 spectra for the two 1G-DEG compounds identified in Oman fluid samples (**Figure S5**) show no fragments from the ester-linked chain of an alkanediol and are thus consistent with a DEG backbone linkage. Yet, it is possible some 1,2-alkanediol glycolipids are present in sampled fluids but were not abundant enough to be captured by MS2 analysis. As a result, the abundance of 1,2-alkanediols is possibly underrepresented in our analyses. IPLs with 1,2-alkanediol linkages can be produced by various thermophilic bacteria (van der Meer et al., 2002; Yang et al., 2006), but the only known producer in Samail Ophiolite fluids is *Meiothermus*, which has only been reported to produce 1,2-alkanediol linkages in N-acetyl glucosaminyl-containing IPLs with three aliphatic chains as a minor component of their lipidome (Ferreira et al., 1999; Yang et al., 2006).

# Supplementary Figures


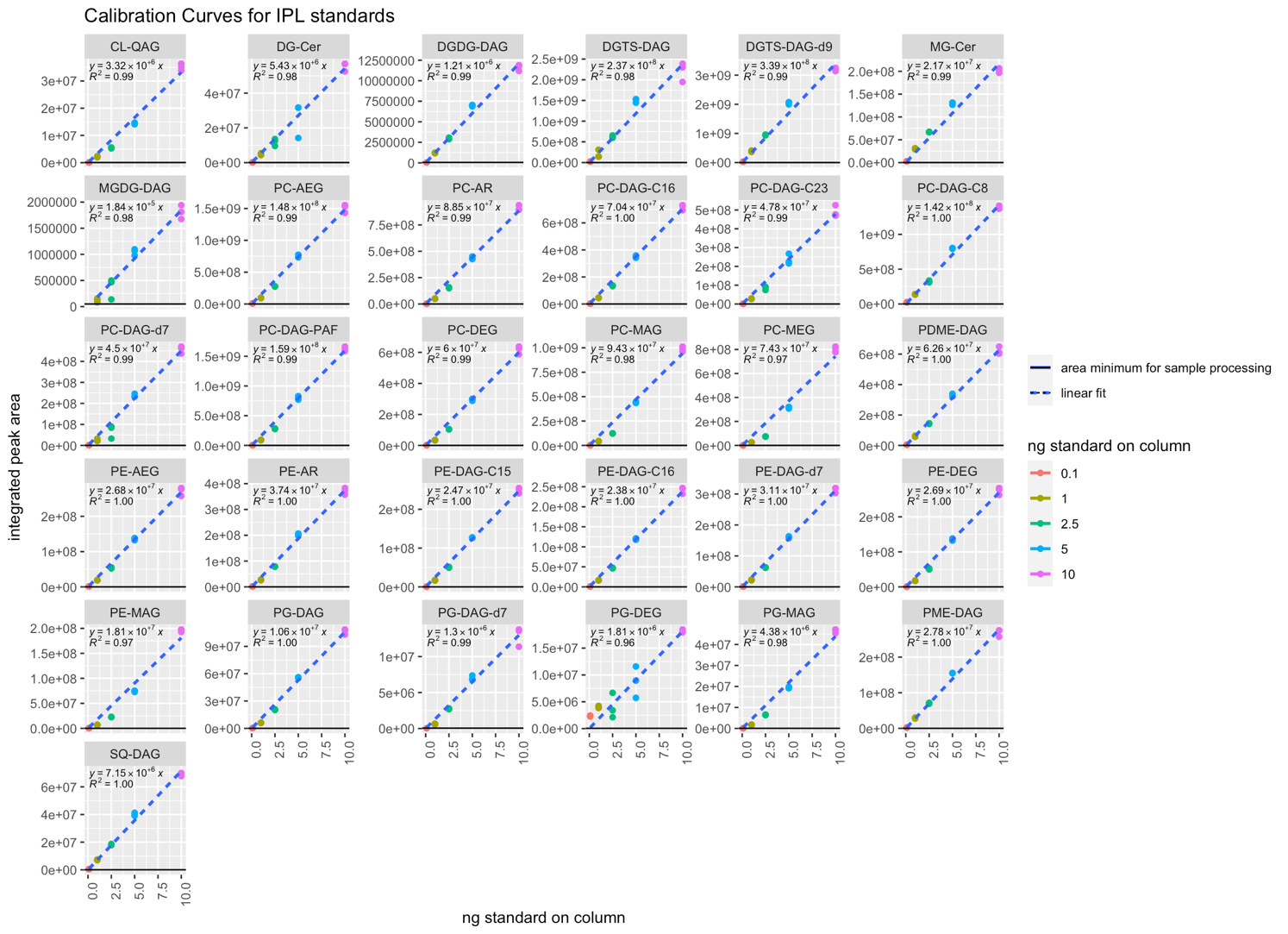


Figure S1: Calibration curves and response factors for IPL standards.


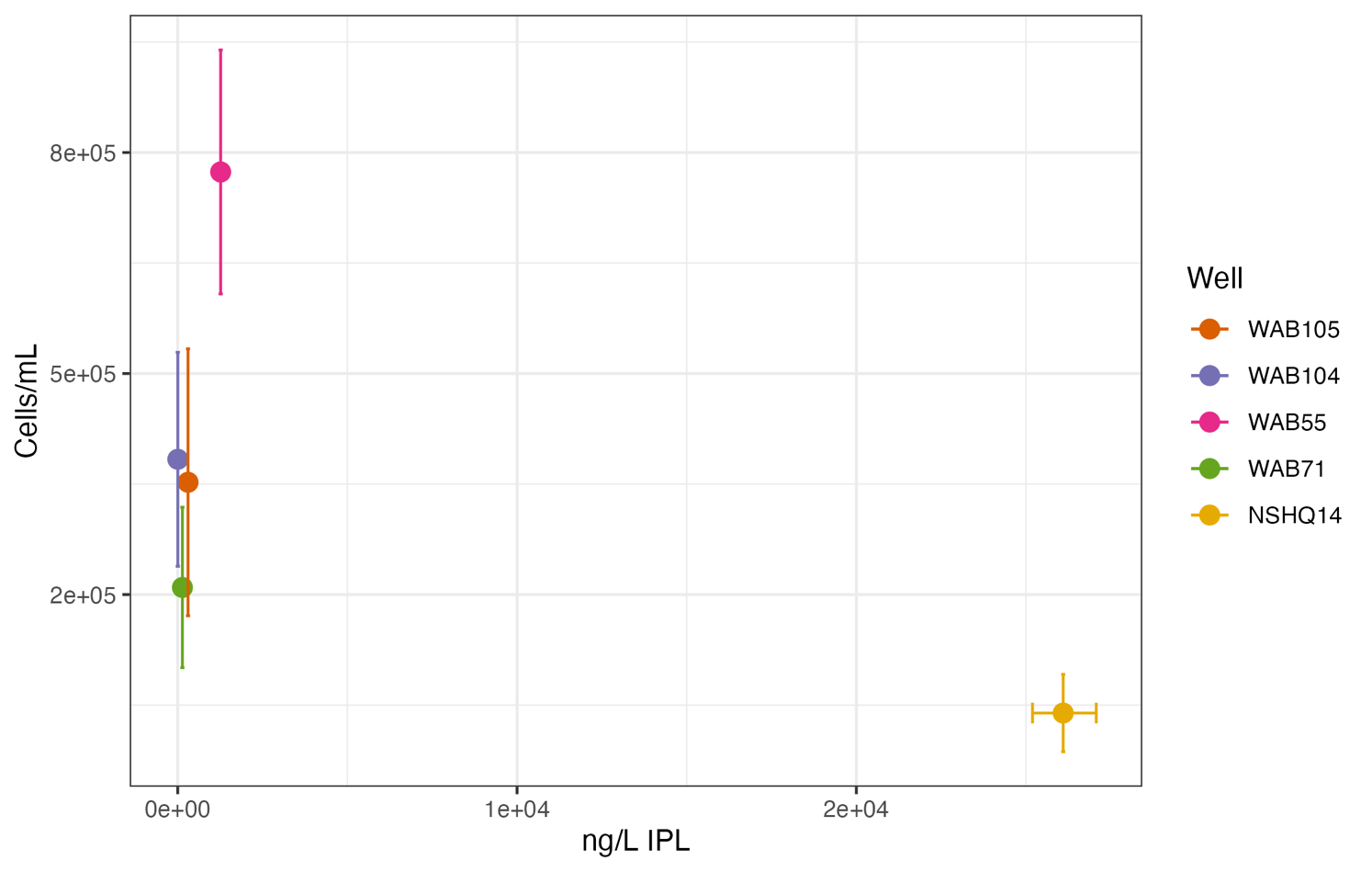


**Figure S2:** No correlation was observed between the plankontic cell abundances reported in Fones et al. 2019 and calculated abundances (ng/L) of IPLs in this study.


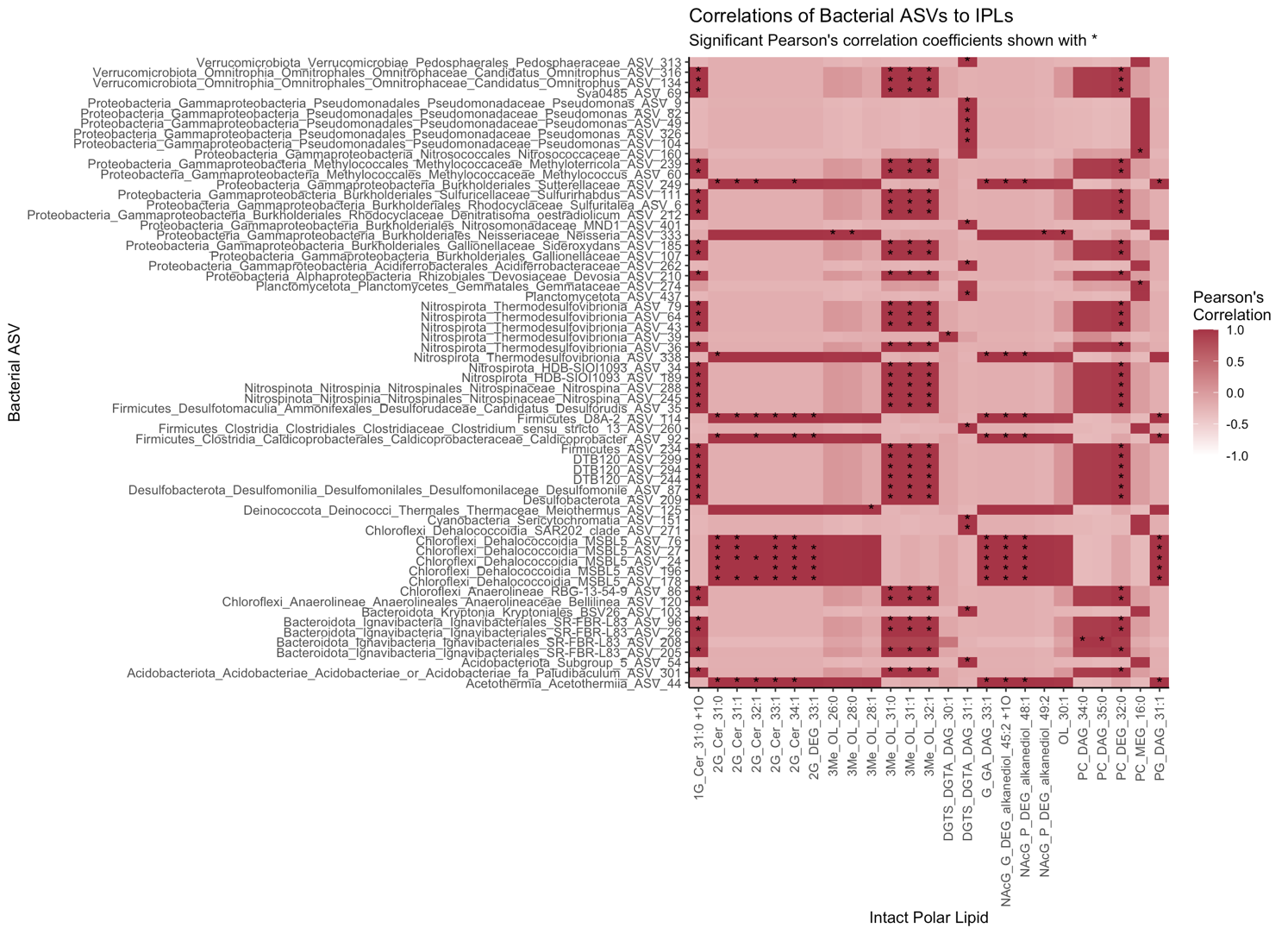


**Figure S3:** Heatmap of Pearson’s correlations of bacterial 16S amplicon sequence variant relative abundance with IPL relative abundance; only IPLs and ASVs with at least one significant correlation are plotted. Significant Bonferroni corrected p-values ( p <.005) are denoted with *.


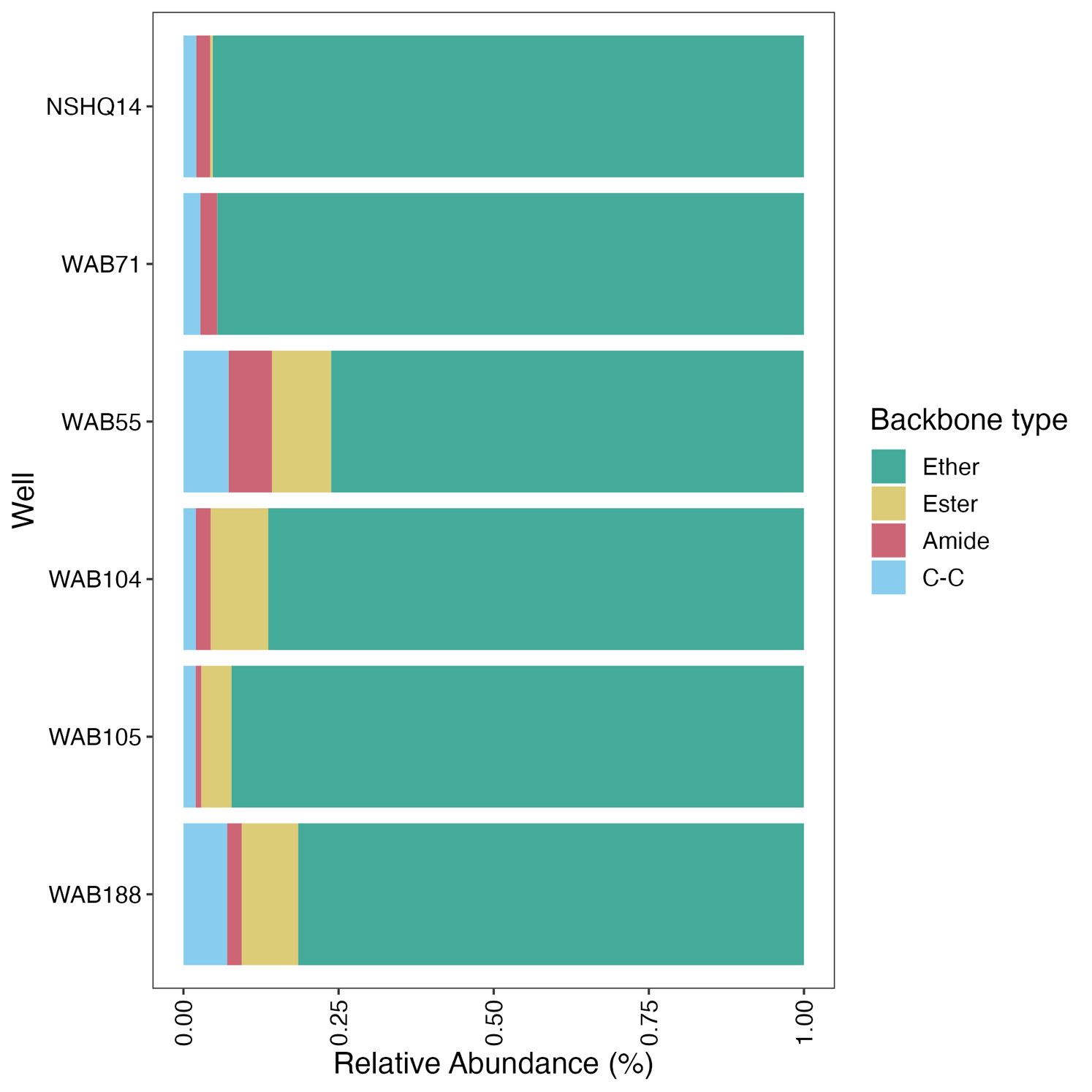


Figure S4: Relative abundances of backbone linkage types.


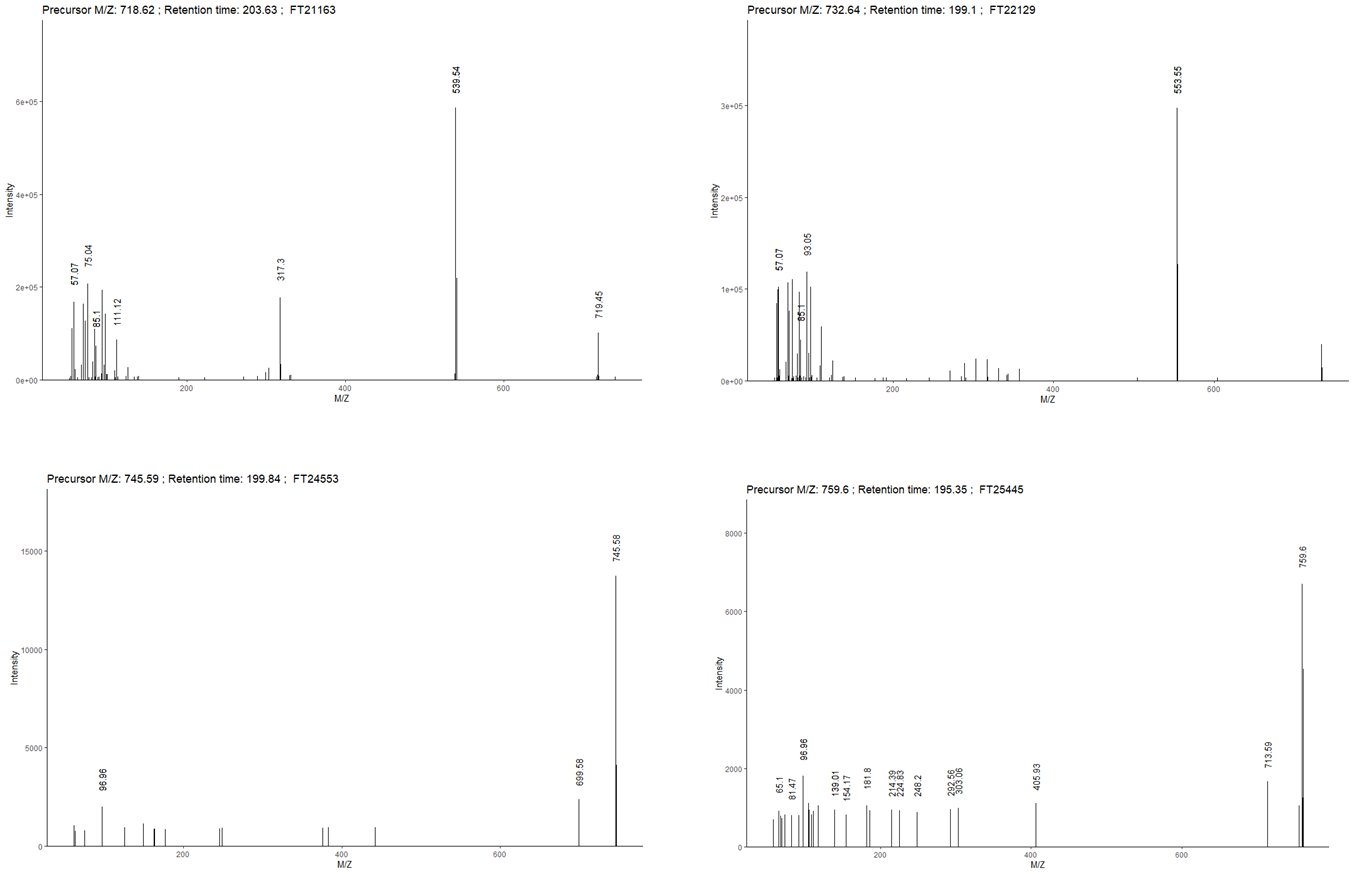
Figure S5: MS2 spectra for identified 1G-DEG IPLs that show a diagnostic loss of 179 Da for a glycosyl headgroup + ammonium adduct as positive ions and no diagnostic fragmentation as negative ions.

# Supplemental References

Ferreira, A.M., Wait, R., Nobre, M.F., Costa, M.S. da, 1999. Characterization of glycolipids from Meiothermus spp. Microbiology (Reading) 145 ( Pt 5), 1191–1199. https://doi.org/10.1099/13500872-145-5-1191

van der Meer, M.T., Schouten, S., Hanada, S., Hopmans, E.C., Damsté, J.S., Ward, D.M., 2002. Alkane-1,2-diol-based glycosides and fatty glycosides and wax esters in Roseiflexus castenholzii and hot spring microbial mats. Arch Microbiol 178, 229–237. https://doi.org/10.1007/s00203-002-0449-8

Yang, Y.-L., Yang, F.-L., Jao, S.-C., Chen, M.-Y., Tsay, S.-S., Zou, W., Wu, S.-H., 2006. Structural elucidation of phosphoglycolipids from strains of the bacterial thermophiles Thermus and Meiothermus. J. Lipid Res. 47, 1823–1832. https://doi.org/10.1194/jlr.M600034-JLR200
